# Molecular Evolution and Functional Characterization of *Drosophila* Insulin-Like Peptides

**DOI:** 10.1371/journal.pgen.1000857

**Published:** 2010-02-26

**Authors:** Sebastian Grönke, David-Francis Clarke, Susan Broughton, T. Daniel Andrews, Linda Partridge

**Affiliations:** 1 Institute of Healthy Ageing, Department of Genetics, Evolution, and Environment, University College London, London, United Kingdom; 2 European Bioinformatics Institute, Wellcome Trust Genome Campus, Cambridge, United Kingdom; University of California San Francisco, United States of America

## Abstract

Multicellular animals match costly activities, such as growth and reproduction, to the environment through nutrient-sensing pathways. The insulin/IGF signaling (IIS) pathway plays key roles in growth, metabolism, stress resistance, reproduction, and longevity in diverse organisms including mammals. Invertebrate genomes often contain multiple genes encoding insulin-like ligands, including seven *Drosophila* insulin-like peptides (DILPs). We investigated the evolution, diversification, redundancy, and functions of the DILPs, combining evolutionary analysis, based on the completed genome sequences of 12 *Drosophila* species, and functional analysis, based on newly-generated knock-out mutations for all 7 *dilp* genes in *D. melanogaster*. Diversification of the 7 DILPs preceded diversification of *Drosophila* species, with stable gene diversification and family membership, suggesting stabilising selection for gene function. Gene knock-outs demonstrated both synergy and compensation of expression between different DILPs, notably with DILP3 required for normal expression of DILPs 2 and 5 in brain neurosecretory cells and expression of DILP6 in the fat body compensating for loss of brain DILPs. Loss of DILP2 increased lifespan and loss of DILP6 reduced growth, while loss of DILP7 did not affect fertility, contrary to its proposed role as a *Drosophila* relaxin. Importantly, loss of DILPs produced in the brain greatly extended lifespan but only in the presence of the endosymbiontic bacterium *Wolbachia*, demonstrating a specific interaction between IIS and *Wolbachia* in lifespan regulation. Furthermore, loss of brain DILPs blocked the responses of lifespan and fecundity to dietary restriction (DR) and the DR response of these mutants suggests that IIS extends lifespan through mechanisms that both overlap with those of DR and through additional mechanisms that are independent of those at work in DR. Evolutionary conservation has thus been accompanied by synergy, redundancy, and functional differentiation between DILPs, and these features may themselves be of evolutionary advantage.

## Author Summary

The insulin/IGF signalling (IIS) pathway plays key roles in growth, metabolism, reproduction, and longevity in animals as diverse as flies and mammals. Most multicellular animals contain multiple IIS ligands, including 7 in the fruit fly *Drosophila melanogaster* (DILP1-7), implying that the diverse functions of IIS could in part be mediated by the functional diversification of the ligands. Although *Drosophila* is a prime model organism to study IIS, knowledge about the function of individual DILPs is still very limited due to the lack of gene-specific mutants. Therefore, we generated mutants for all 7 *dilp* genes and systematically analyzed their phenotypes. We show that loss of DILP2 extends lifespan and describe a novel role for DILP6 in growth control. Furthermore, we show that DILPs are evolutionary conserved and can act redundantly, supporting the hypothesis that functional redundancy itself can be of evolutionary advantage. We also describe a specific interaction between IIS and the endosymbiontic bacterium *Wolbachia* in lifespan regulation. This finding has implications for all longevity studies using IIS mutants in flies and offers the opportunity to study IIS as a mechanism involved in host/symbiont interactions. Finally, we show that DILPs mediate the response of lifespan and fecundity to dietary restriction.

## Introduction

The ability of organisms to respond appropriately to changes in their environment is key to survival and reproductive success. An essential environmental variable for all organisms is their food supply and energetically demanding processes, such as growth, metabolism and reproduction are matched to nutrition by nutrient-sensing pathways, such as the insulin/IGF signalling (IIS) and TOR pathways [1]. An important recent discovery has been that reduced activity of IIS and TOR can slow aging and increase stress resistance and lifespan in the yeast *Saccharomyces cerevisiae*, the nematode worm *Caenorhabditis elegans*, the fruit fly *Drosophila melanogaster* and mice [2]. The mechanisms by which these pathways exert their diverse effects are hence of interest, as are the ways in which these parallel biological roles are achieved in evolutionarily diverse organisms.

The IIS pathway includes both peptide ligands, which can act at a distance, and intracellular components. In mammals, the ligands include insulin, the insulin-like growth factors (IGF) and relaxins. IGFs are mainly involved in growth control during development, whereas insulin secretion from pancreatic β-cells controls carbohydrate and lipid metabolism. Relaxins are produced by the ovary and are involved in reproduction. Insulin-like peptides (ILPs) have also been identified across a broad range of invertebrates, including molluscs, the nematode *Caenorhabditis elegans* and several insect species [3]. Most invertebrate genomes contain multiple ILPs, including 40 in *C. elegans* [4] and 7 in *Drosophila melanogaster* (DILP1-7) [5]. In contrast, while mammals often have up to 4 isoforms of the cellular components of IIS, they are encoded by single genes in *Drosophila*, including one *Drosophila* Insulin receptor (DInR), one insulin receptor substrate (*chico*) and one downstream forkhead box O transcription factor (dFOXO) [1]. The relative simplicity of the cellular IIS pathway, together with the diversification of DILPs, implies that the diverse functions of IIS could be in part mediated by functional diversification of the ligands.

Supporting functional differentiation between ligands, each *dilp* gene shows a characteristic spatio-temporal expression pattern. For instance, DILP4 is expressed in the embryonic midgut and mesoderm [5], DILP6 predominantly in the larval and adult fat body, with expression strongly up-regulated in the transition from larva to pupa [6]. DILP7 is expressed in specific neurons that innervate the female reproductive tract [7],[8], and inactivation of them results in sterile flies with an “egg-jamming” phenotype, suggesting that DILP7 could be a *Drosophila* relaxin [7]. DILP1, 2, 3 and 5 are expressed in brain median neurosecretory cells (MNCs) of the larval brain [5],[9],[10], but only DILP2, 3 and 5 could be detected in MNCs of the adult fly [11]. DILP2 is also expressed during development in imaginal discs, and in salivary glands and DILP5 in follicle cells of the female ovary [5].

Targeted ablation of the MNCs during early larval development results in developmental delay, growth defects and elevated carbohydrate levels in the larval hemolymph [10], while later ablation, during the final larval stage, results in lower female fecundity, increased storage of lipids and carbohydrates, elevated resistance to starvation and oxidative stress and increased lifespan [11]. Notably, expression of ILPs in MNC is evolutionarily conserved among insects, suggesting important evolutionarily conserved functions for these cells and their ligands. Furthermore, similar developmental programs are involved in the specification of MNCs in *Drosophila* and pancreatic beta cells in mammals, suggesting that insulin-producing cells of invertebrates and vertebrates may be derived from a common ancestry [12]. It is not yet clear if the phenotypes of MNC-ablated flies result from loss of one or more of the DILPs, or whether MNCs have functions independent of DILPs. Nor is it known if MNC-expressed DILPs have specific functions or act redundantly.

All seven DILPs possess the ability to promote growth, with DILP2 the most potent, and these ligands therefore probably all act as DInR agonists [9]. Over-expression of DILP2 also suppressed germ line stem cell loss in ageing females, probably through action in the stem cell niche [13]. DILP2 may also modulate lifespan, because its transcript is lowered in various mutant, long-lived flies. Over-expression of dFOXO in the adult fat body [14], over-expression of a dominant negative form of p53 in MNCs [15], increased JNK activity in MNCs [16] and hypomorphic mutants of the *Drosophila* NPY like protein sNPF [17], have all been reported to both extend lifespan and reduce the level of DILP2 expression. However, direct reduction in the level of DILP2 by *in vivo* double-stranded RNA interference (RNAi) did not increase lifespan [18], leaving the role of DILP2 unclear.

Dietary restriction (DR), a reduction in food intake without malnutrition, extends lifespan in diverse animals. In both *C. elegans* and *Drosophila* extension of lifespan by DR has been suggested to be independent of IIS, because lifespan increases in response to DR in animals lacking the key IIS effector FOXO transcription factor [19]–[21]. However, the finding could indicate instead that, in the absence of a normal increase in FOXO activity during DR, other pathways can act redundantly to increase lifespan. Indeed, other evidence has suggested that reduced IIS and DR may extend lifespan through overlapping mechanisms [19],[22]. DILP3 and DILP5 transcript levels are reduced in starved larvae [9] and DILP5 in DR adult flies [20], potentially indicating a role of DILPs in the fly's response to DR.

Gene families generally expand by gene duplication, and the duplicate copies are retained either if there is a requirement for large amounts of the gene product or if the duplicate copies undergo some sequence divergence and functional differentiation [23]. Recent work has suggested that feedback between partially redundant duplicate genes could itself be an important source of information aiding signal transduction [24]. It is not known if, as well as the functional differentiation implied by the findings described above, there is functional redundancy between the *Drosophila* ILPs. Nor is it known if there is stability of sequence differentiation and of membership of this gene family over evolutionary time or whether there is gene turnover. In addition, assignment of specific functions to individual *dilp* genes requires experimental manipulation. Although studies using RNAi have been informative [17],[18], RNAi can be prone to off-target effects [25] and other forms of cellular toxicity, and often results only in hypomorphic phenotypes.

We have used the completed genome sequences of 12 *Drosophila* species [26] to examine the stability of the *dilp* gene family, and found that the 7 DILPs have remained present and clearly differentiated from each other in sequence over a period of 40–60 million years. Evolutionary conservation of different regions of the peptides suggests that 6 of the 7 DILPs are cleaved like mammalian insulins, while the seventh may remain uncleaved, like mammalian IGFs. We generated specific null mutants for all seven DILPs, by homologous recombination or P-element mediated excision, and also generated flies that lack two or more DILPs simultaneously. Using these mutants we made a systematic analysis of DILP function in development, metabolism, reproduction, stress resistance, lifespan and response to DR of *Drosophila*. We show that DILPs can act redundantly, which suggests that redundancy among ILPs may be of evolutionarily advantage. We found both synergy and compensation of expression between DILPs. In particular DILP6 in the fat body compensated for the loss of MNC DILPs, demonstrating that DILPs are part of a complex feedback system between the central nervous system and peripheral tissues such as the fat body, which controls development, metabolism and reproduction. We further show that DILP2 is an important determinant of lifespan, describe a novel role for the fat body derived DILP6 peptide in growth control and demonstrate that *dilp7* null mutants have normal fecundity, contrary to the suggestion DILP7 could be a *Drosophila* relaxin. Finally, we describe a specific interaction between the endosymbiont *Wolbachia* and IIS in the regulation of lifespan and show that DILPs mediate the responses of lifespan and fecundity to DR in *Drosophila*.

## Results

### Phylogenetic analysis of ILPs in the genus *Drosophila*

**Figure 1:**
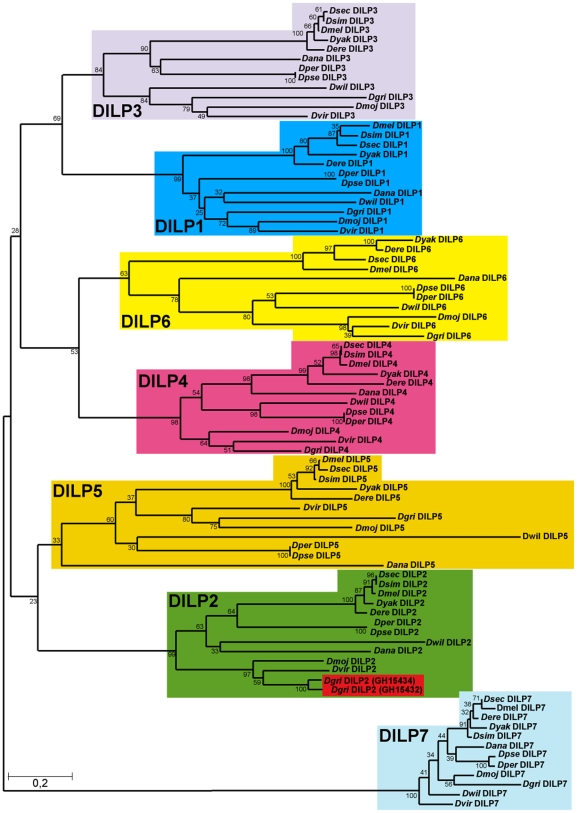
Phylogeny of Drosophila insulin-like peptides. The evolutionary history of the seven DILP families from 12 fully sequenced Drosophila species inferred using the Neighbour-Joining method. Bootstrap replicate support percentages are shown next to the branches. The tree is drawn to scale, with branch lengths proportional to evolutionary distance computed using the JTT matrix-based method. 7 DILPs have remained present and clearly differentiated from each other during the more than 40 million years of evolution of *Drosophilidae* flies. *D. grimshawi* contains two DILP2 peptides (shaded in red).

Taking advantage of the 12 sequenced *Drosophila* genomes [26], we investigated the evolution of ILPs across the *Drosophila* genus. As in *D. melanogaster*, seven *dilp* genes were identified in all 12 *Drosophila* species, with the exception of *D. simulans* and *D. grimshawi* (Figure 1). Using the Jones-Thornton-Taylor (JTT) matrix to calculate average amino acid distances, we found that the distances between the seven DILPs within each species is much greater than the distances among the putative orthologues in the different *Drosophila* species (Figure 1) We did not identify a *dilp6* gene in *D. simulans*, probably because of sequencing gaps in the corresponding genomic region. Thus the seven *dilp* genes were present before the divergence of the 12 *Drosophila* species more than 40 million years ago, and none has been turned over during that period. Interestingly, the *D. grimshawi* genome contains eight *dilp* genes, as a result of a duplication of *dilp2* (Figure 1). *D. grimshawi* is a member of the endemic Hawaiian *Drosophila* species, which are characterized by their large body size. The finding that DILP2 is an important regulator of growth in *D. melanogaster* ([9] and see below) suggests that evolutionary changes in *dilp2* gene expression may have contributed to the evolution of body size in the Hawaiian *Drosophila* species.

In mammals, IIS ligands are synthesized as pre-propeptides, consisting of a signal peptide and contiguous B-C-A peptides. In insulin and relaxin the C-peptide is clipped out by a convertase enzyme targeting basic amino acid cleavage sites, to produce a bioactive peptide consisting of A- and B-chain linked by 2–3 disulfide bridges. In contrast, IGFs contain a shortened C-peptide, which is not removed, resulting in a single chain peptide hormone. We therefore examined evolutionary conservation of different regions of the DILPs across the 12 *Drosophila* species to determine if the proteins are likely to be cleaved. Amino acid alignments of DILP pre-propeptides from the different *Drosophila* species shows that the bioactive peptides, i.e. the A and B chains are more conserved than the signal peptides and the C-peptides (Figure S1). The only exception is the shortened C-peptide in DILP6, which shows a similar degree of conservation to the A and B chains. This finding may indicate that although it contains a basic cleavage site the C-peptide is not removed and is part of a single chain bioactive DILP6 peptide, as has been recently suggested for the DILP6-like BIGFLP protein in the silkworm *Bombyx mori* [6]. Thus, DILP6 may resemble IGF rather than insulin. Despite the low amino acid conservation, functional signal peptides were found to be present in all DILP pre-propeptides using the Signal P prediction software [27], which indicates that all seven DILPs act as secreted peptide hormones in all 12 *Drosophila* species. Furthermore, cysteine residues involved in disulfide bridge formation as well as basic cleavage sites are highly conserved (Figure S1A), further supporting the view that DILPs, except for DILP6, resemble insulin and consist of heterodimeric peptides of A and B-chain linked by disulfide bridges.

Although the seven DILPs have been stably differentiated and retained in the 12 *Drosophila* genomes, they show different degrees of amino acid conservation (Figure S1B), suggesting different degrees either of functional constraint or of directional selection. DILP7 is by far the most highly conserved DILP peptide, with an overall amino acid identity of 76% between the pre-propeptides of *D. melanogaster* and the most distantly related *Drosophila* species *D. grimshawi*, increasing to 83% and 94%, respectively, when only the A and B chains are considered (Figure S1B). Indeed, only DILP7 has *bona fide* orthologues outside the *Drosophila* family, with 64–66% amino acid identity (A and B chain, 48% overall) with ILP5 from the mosquitoes *Anopheles gambiae* and *Aedes aegypti* and 63% and 55% (A and B chain, 48% overall) with ILP7 from the red flour beetle *Tribolium castaneum* [28],[29]. DILP4 is next most conserved, with only 52% overall amino acid identity and 75% and 70% identity in the A and B chain, respectively (*D. melanogaster* vs. *D. grimshawi*). DILPs 1, 2, 3, 5 and 6 evolved faster than DILP4 and DILP7 and at about the same rate as each other (ca. 40% overall amino acid identities, 50–55% identities within the A and B chain, *D. melanogaster* vs. *D. grimshawi*, Figure S1B). The higher amino acid sequence conservation of DILPs 7 and 4 suggests that these two DILPs might carry essential functions that are different from the other DILPs and can therefore not be compensated by the other DILPs.

### Generation of *Drosophila* insulin-like peptide mutants

**Figure 2:**
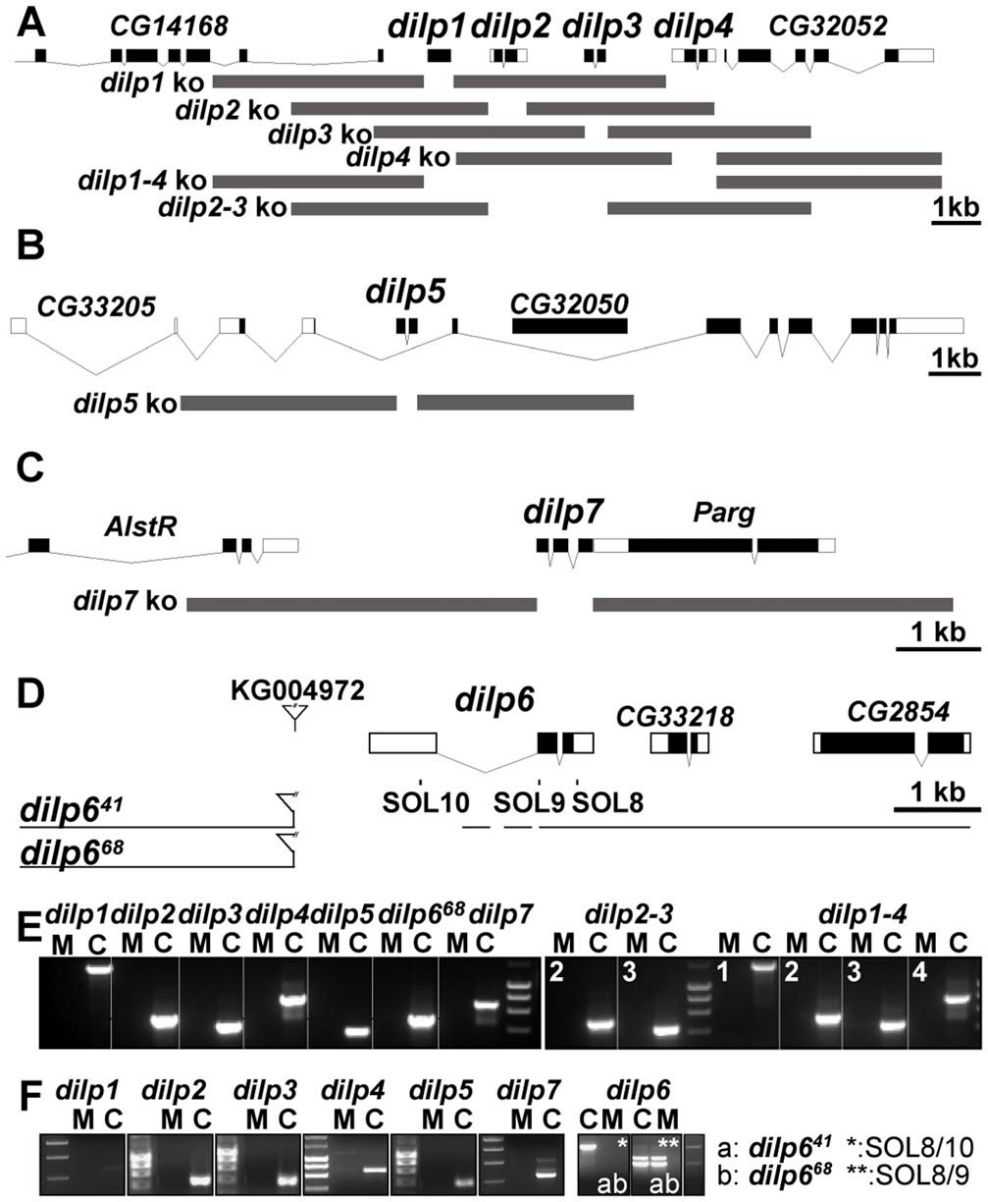
Gene locus organization and generation of *dilp* mutants. (A–C) Null mutations for *dilp1, 2, 3, 4, 5, 7, 2*–*3*, and *1*–*4* were generated by ends-out homologous recombination and by imprecise P-element excision for *dilp6* (D). (A) The *dilp1*–*4* genes cluster at cytological position 67C8 is separated from *dilp5* by two intervening genes of unknown function (Flybase: *CG32052* and *CG33205*). (B) *dilp5* is located within an intron of the *CG33205* gene. (C) *dilp7* is located on the X-chromosome at 3E2 immediately downstream of the essential *Poly(ADP-ribose) glycohydrolase* (*Parg*) gene. Donor constructs (dilp ko) for ends-out homologous recombination are indicated by grey bars. Gap between grey bars indicates the genomic region replaced by a *white^hs^* marker gene. Coding parts of exons are marked in black, non-coding parts by white boxes. (D) Transposon integration line KG004972 was used to generate *dilp6* deletion mutants *dilp6^41^* and *dilp6^68^*. Note: both *dilp6* deletion alleles contain remaining P-element sequence, hatched line: region of breakpoint in *dilp6^41^*. (E) PCR on genomic DNA of *dilp* mutants with *dilp* specific primer combinations shows homologous recombination mediated replacement of *dilp* genes by the *white^hs^* marker gene and deletion of *dilp6* in *dilp6^68^* mutants (M: mutant, C: control). (F) RT–PCR analysis confirms that *dilp* mutants are transcript null alleles. *dilp6^41^* mutants express ectopic *dilp6* transcripts that lack the first exon but contain the full ORF. *dilp6^68^* mutants are *dilp6* transcript null alleles.

In order to analyse the *in vivo* function of individual *dilp* genes, we generated *dilp*-specific mutants by ends-out homologous recombination [30] for *dilp1*, *2*, *3*, *4*, *5* and *7* (Figure 2A–2C) and by P-element mediated imprecise excision for *dilp6* (Figure 2D). In addition, to address redundancy and synergy among individual *dilp* genes, we used homologous recombination to generate mutant flies with a combined knock out of two or several DILPs, including *2*, *3* (*dilp2–3*), *2*, *3*, *5* (*dilp2–3,5*), *1*, *2*, *3*, *4* (*dilp1-4*) and *1*, *2*, *3*, *4*, *5* (*dilp1–4,5*).

**Figure 3:**
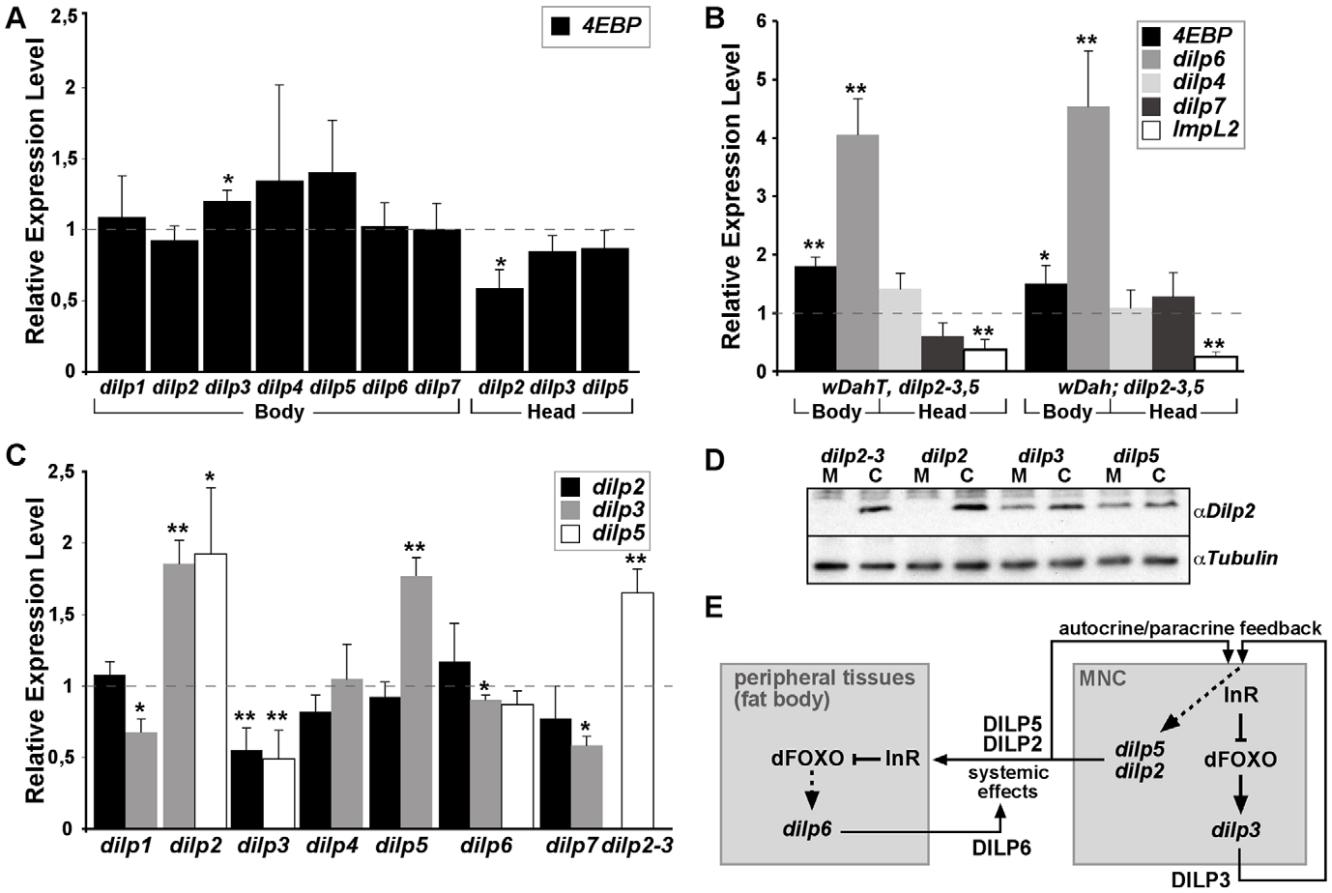
Compensatory regulation of gene expression in *dilp* mutants. (A) Q-RT-PCR analysis of *4E-BP* expression on fly bodies and fly heads of *dilp* single mutants. Expression of the IIS downstream target gene *4E-BP* is not changed in *dilp* single mutants. (B) Q-RT-PCR analysis demonstrates up-regulation of *4E-BP* and *dilp6* and down-regulation of *ImpL2* in *dilp2–3,5* mutants independent of *Wolbachia* infection status (w^DahT^: *Wolbachia*-, w^Dah^: *Wolbachia*+). (C) Compensatory regulation among MNC-expressed DILPs. Expression levels of DILP2, 3, and 5 were measured by QRT-PCR on RNA extracted from heads of *dilp* mutants. (D) Western blot analysis of DILP2 expression in total head protein confirms lack of DILP2 expression in *dilp2* and *dilp2*–*3* mutants and downregulation of DILP2 levels in *dilp3* mutants. M: homozygous mutant, C: *w^111^8* control. An anti-Tubulin antibody was used as loading control. (E) Diagram summarizing the feedback system involved in the control of DILP expression levels. DILP3 is part of a feedback system that acts in an autocrine/paracrine manner to regulate DILP expression in the MNC. DILP expression between MNC in the central nervous system and the peripheral fat body tissue is regulated by a negative feedback system that involves DILP6. See text for further details. Arrows denote activation, blunted lines denote repression. All experiments in (A–D) were done using 8–10 day old females reared on 1x SY-A food. Expression level of mutants were normalised to the corresponding wild type control, which by default was set to 1. * p<0.05, ** p<0.01.

Ends out homologous recombination donor constructs were designed to delete the complete coding sequence of the targeted *dilp* gene, by replacing it with a *white* marker gene without affecting the genomic sequence of adjacent genes (Figure 2A–2C). Putative homologous recombination events were tested by PCR on genomic DNA with gene-specific primer combinations for the insertion of the *white* marker gene into the target gene location (Figure 2E). Several independent targeting events were recovered per *dilp* gene. Long-range PCR analysis on genomic DNA showed that most targeting events were precise homologous recombinations (Figure S2 and data not shown) and only these were used for subsequent experiments. Reverse Transcription (RT) PCR analysis showed that the *dilp* mutants are transcript-null alleles (Figure 2F). Lack of DILP expression in the mutants was confirmed by immunohistochemistry on adult fly brains (Figure S3) and by Western blot analysis (Figure 3D). The single mutants were specific for individual *dilp* genes. For instance, *dilp2* mutants lacked expression only of DILP2, not of DILP3 or DILP5 (Figure S3D, S3E, S3F).

*dilp6* mutant flies were generated by imprecise P-element excision of KG04972 integrated in the 5′upstream region of the *dilp6* gene (Figure 2D). Two different *dilp6* alleles were isolated, the small deletion *dilp6^41^* covering the *dilp6* 5′upstream region including the first exon and the large deletion *dilp6^68^* covering the complete *dilp6* gene as well as at least 4 other genes. RT PCR analysis confirmed *dilp6^68^* to be a transcript null allele (Figure 2F). *dilp6* expression was still detectable in *dilp6^41^* mutants. However, 5′ RACE analysis revealed that these transcripts lacked the first exon of the dilp6 gene and instead contained ectopic sequence including additional ORFs immediately upstream of the *dilp6* ORF, which might interfere with the translation of the DILP6 peptide (for details see Text S1). Consistently, whenever tested both *dilp6* mutant alleles were phenotypically indistinguishable from each other. In addition, *dilp6^41^* mutant flies showed the same developmental growth defects as flies in which *dilp6* is knocked down by RNAi [31], suggesting *dilp6^41^* to be a strong hypomorph or even a functional null allele.

### Compensatory regulation

Reduced expression of DILPs has been associated with changes in IIS pathway activation [15]. We therefore evaluated IIS activity in body tissues of adult *dilp* mutants by measuring transcript levels of the translational regulator 4E-BP (encoded by *Thor*), a direct target of dFOXO, which is induced when IIS is repressed and dFOXO is activated (Figure 3A and 3B). As expected, *4E-BP* transcript levels were up-regulated in *dilp2*–*3,5* mutants (Figure 3B), consistent with peripheral activation of dFOXO and reduced IIS. However, we did not observe significant up-regulation of *4E-BP* transcript levels in *dilp* single mutants, except for *dilp3* mutants, which showed slight up-regulation of *4E-BP* levels in bodies but not in heads (Figure 3A). Thus, the knock out of most individual DILPs did not result in systemic down-regulation of IIS that was detectable by measuring *4E-BP* transcript levels. A possible explanation for this finding could be that DILPs act redundantly as part of a negative feedback system in which knock-out of one DILP is compensated by the up-regulation of others.

To address the possibility of compensatory regulation we measured *dilp* transcript levels in *dilp* mutant flies (Figure 3B and 3C). We did not detect any up-regulation of *dilp2* or *dilp3* transcript levels in *dilp1, 4, 6* or *7* mutants, however, *dilp5* was up-regulated in *dilp2* and *dilp2*–*3* mutants and *dilp3* was up-regulated in *dilp2* and *dilp5* mutants (Figure 3C), demonstrating compensatory transcriptional regulation among MNC-expressed DILPs. Intriguingly, *dilp2* and *dilp5* expression was down-regulated in *dilp3* mutants (Figure 3C and 3D), suggesting synergy in expression, with *dilp3* acting as a positive regulator of *dilp2* and *dilp5* expression. Remarkably, while expression of *dilp4* and *dilp7* was not significantly changed in *dilp2*–*3,5* mutants, the fat-body-expressed *dilp6* gene was strongly up-regulated (Figure 3B), suggesting the existence of a negative feedback system that acts to coordinate DILP expression between the MNCs in the central nervous system and peripheral tissues like the fat body (Figure 3E). Interestingly, expression of the DILP binding protein ImpL2, a negative regulator of IIS [32], was down-regulated in *dilp2*–*3,5* mutants (Figure 3B), demonstrating that the negative feedback system is not restricted to the regulation of *dilp* transcription.

### Systematic analysis of DILP function

The compensatory regulation among *dilp* genes may indicate that they act at least in part redundantly. However, each *dilp* gene is expressed in a tissue- and stage specific manner, suggesting that there is also diversification of their functions. In order to examine the *in vivo* function of individual *dilp* genes and to address whether and to what extent they act redundantly and synergistically to execute these functions, we initiated a systematic analysis of the *dilp* mutants, addressing phenotypes associated with MNC-ablation and reduced IIS, including egg-to-adult survival, development time, organismal growth, stress resistance, energy storage, lifespan and fecundity (summarized in Table 1).

**Table 1:** Systematic analysis of DILP function.

Mutant	Viability	Dev. time	Body weight	Median lifespan	Lifetime fecundity	Paraquat resistance	Starvation resistance	Lipid	Glycogen	Trehalose
*dilp1*	100%	NC	m: −7%** f: −7%**	NC	NC	NC	NC	NC	NC	ND
*dilp2*	100%	m: +8h f: +17h	m: −5%** f: −11%**	m: +9%** f: +8–13%**	−25%**	NC	NC	NC	NC	+ 64%*
*dilp3*	100%	NC	NC	NC	−22%*	NC	NC	NC	NC	NC
*dilp4*	100%	NC	NC	NC	NC	NC	NC	NC	NC	ND
*dilp5*	100%	NC	NC	NC	−18% (P<0,07)	NC	NC	NC	NC	NC
*dilp6^41^*	100%	m: +4h	m: −10%** f: −20%**	NC	−46%**	m: NC	m: NC	+21% *	NC	ND
*dilp6^68^*	100%	m: +4h	m: −13%** f: −20%**	ND	ND	m: NC	m: NC	ND	ND	ND
*dilp7*	100%	NC	NC	NC	NC	NC	NC	NC	NC	ND
*dilp2–3*	100%	ND	m: −7%** f: −7%**	f: +12% **	−27%**	NC	NC	ND	ND	ND
*dilp1–4*	100%	f: +25h	m: −13%** f: −11%**	NC	−14%*	+21%*	+18%*	ND	ND	ND
*dilp2–3,5*	100% f 60% m	m: +10−17d f: +8−17d	f: −42%**	NC	−69%**	+25%**	NC	+19% **	+72%**	ND
*dilp1–4, 5*	<100%	m: +10−17d f: +8−17d	f: −53%**	ND	ND	ND	ND	ND	ND	ND
*dilp6^41^; 2–3, 5*	0%	–	–	–	–	–	–	–	–	–
*dilp6^41^; 1*–*4, 5*	0%	–	–	–	–	–	–	–	–	–
*dilp6^68^; 2*–*3, 5*	0%	–	–	–	–	–	–	–	–	–
*dilp6^68^; 1*–*4, 5*	0%	–	–	–	–	–	–	–	–	–
*dilp7; 2*–*3, 5*	100% f 60% m	m: +10−17d f: +8−17d	f: −41%**	ND	ND	ND	ND	ND	ND	ND
*dilp7; 1–4,5*	<100%	m: +10−17d f: +8−17d	f: −52%**	ND	ND	ND	ND	ND	ND	ND

ND, not determined; NC, not changed; f, females; m, males. If not indicated otherwise, all data are for *Wolbachia*-free females. * p<0.05, ** p<0.01.

### Egg-to-adult survival

All seven *dilp* single mutants as well as *dilp2–3* and *dilp1–4* mutants were homozygous viable (Table 1). In contrast, *dilp2–3,5* mutants showed sex-specific lethality; whereas homozygous mutant females showed normal viability, only 50*–*60% of *dilp2–3,5* homozygous males developed into adult flies (Table 1). Survival was not further negatively affected in *dilp7;2–3,5* mutant flies, but was reduced in *dilp1–4,5* mutants. Intriguingly, animals that lacked all DILPs except for DILP6 (*dilp7;1–4,5* mutants) still developed into adult flies. In contrast, combined knock-out of DILPs 2, 3, 5 and 6 caused complete lethality in males and females. This result indicates that DILP6 acts redundantly to MNC-expressed DILPs, consistent with the compensatory up-regulation of DILP6 transcript in *dilp2–3,5* mutants (Figure 2B). Lethality in combination with *dilp2–3,5* mutants was observed for both *dilp6* alleles, further evidence that *dilp6^41^* is a *dilp6* loss-of function allele.

### Development time

**Figure 4:**
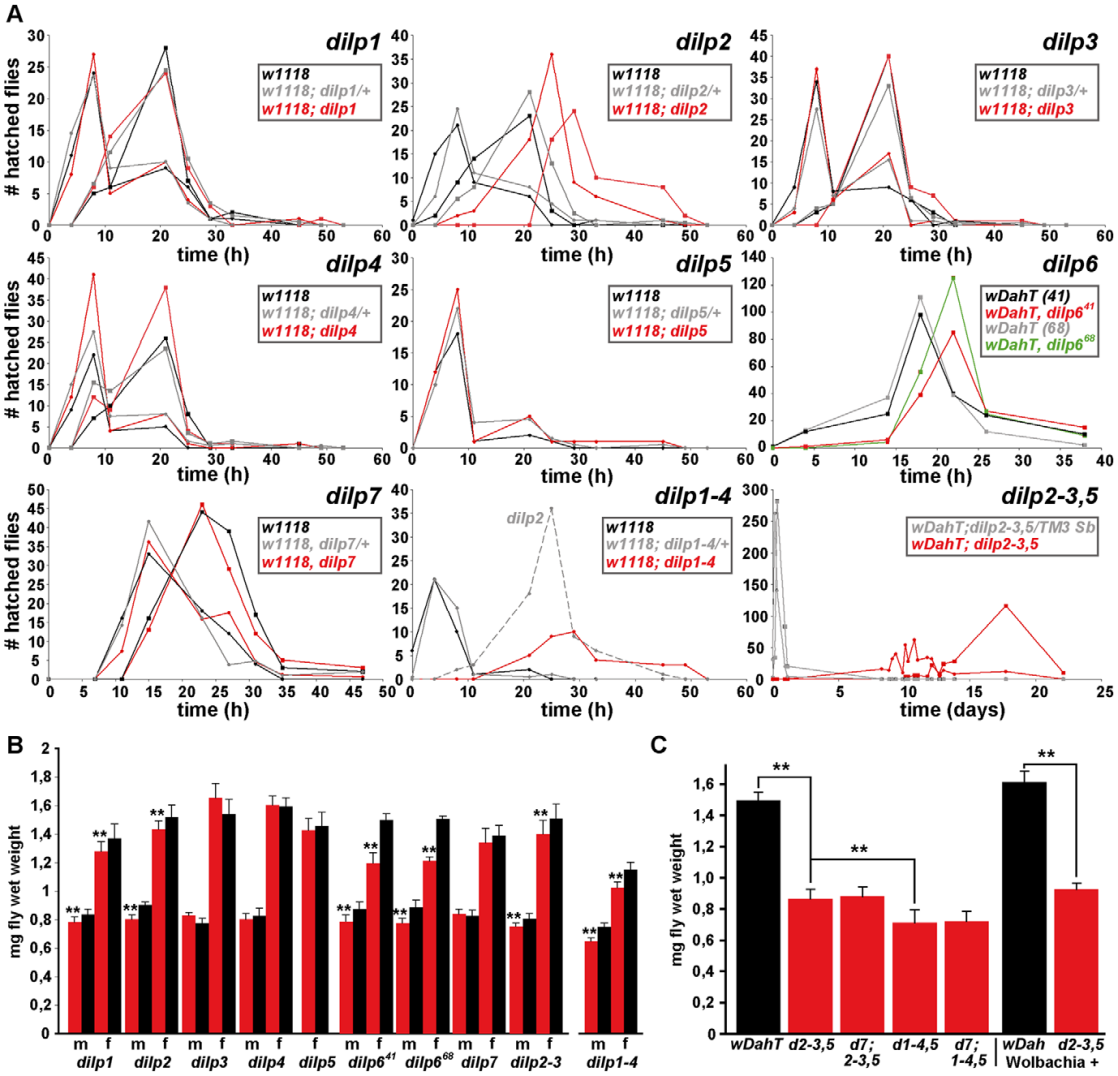
Development time and body weight of *dilp* mutants. (A) Egg-to-adult development time of *dilp* mutants. Only the hatching period of the adult flies is shown. Rectangle: males (m), circle: females (f). (Note: number of heterozygous flies was halved to adjust to the number of homozygous and *w* control flies.) (B) Body weight of *dilp* mutant (red) males (m) and females (f) compared to controls (black). (n = 20 flies). (C) Body weight of female flies that lack multiple *dilp* genes. (n = 40 f; *dilp1–4,5* n = 20 f; *dilp7,1–4,5* n = 18 f). *Wolbachia*+ flies (w^Dah^) weigh more than *Wolbachia*- flies (w^DahT^). Body weight in (B,C): w^DahT^ background, except for *dilp1–4* mutants: *w^1118^*. ** p<0.01, t-test.

*dilp2* and *dilp6* were the only single mutants that showed a delay in egg-to-adult development (Figure 4A, Table 1). Development time was only slightly further delayed in *dilp1–4* mutants compared to *dilp2* single mutants. In contrast, *dilp2–3,5* mutants had a severe developmental delay (Figure 4A), comparable to flies with ablated MNCs or DInR mutants [10]. The developmental delay was caused by delays in larval or pupal development, because *dilp2–3,5* homozygous mutant embryos developed into first instar larvae at the same rate as wild type controls (data not shown). *dilp2–3,5* mutants also eclosed over a much longer period; all control flies eclosed within a day while *dilp2–3,5* mutant flies continued to hatch over a period of almost ten days (Figure 4A). *dilp1–4,5* mutants, *dilp7;2–3,5* mutants and *dilp7,1–4,5* mutants had similar development times to *dilp*2*–*3,5 mutants (Table 1), suggesting that DILPs1, 4 and 7 are not involved in the regulation of developmental timing.

### Organismal growth

Organismal growth was analysed by measuring the body weight of adult flies (Figure 4B and 4C, Table 1). *dilp 3, 4, 5* and *7* single mutants showed normal body weight. *dilp1* and *dilp2* mutants showed a slight reduction in weight (Figure 4B), consistent with the shorter adult body length seen upon RNAi-mediated knockdown of DILP1 and DILP2 [17]. Although *dilp1* mutants weighed less, they developed at the same rate as control flies, demonstrating that growth defects are not necessarily coupled with a delay in development. Intriguingly, *dilp6* mutants showed the biggest reduction in body weight of all *dilp* single mutants (Figure 4B). DILP6 resembles IGFs and is expressed at high levels in the fat body but not in the MNCs [6], suggesting DILP6 to be an IGF-like peptide secreted by the fat body that promotes growth during larval-pupal development.

*dilp2–3* mutants weighed as much as *dilp2* mutants and only a minor additional decrease in body weight was seen in *dilp1–4* mutants (Figure 4B), likely to be the result of the combined lack of DILP1 and DILP2. In contrast, body weight of *dilp2–3,5* mutants was severely reduced (Figure 4C), and an even further reduction was observed in *dilp1–4, 5* mutants, which were approximately 50% smaller than controls (Figure 4C). Lack of DILP7 did not result in a further decrease in body weight of either *dilp2–3,5* or *dilp1–4,5* mutants (Figure 4C), suggesting that DILP7 does not contribute to the regulation of organismal growth.

### Stress resistance and energy storage

Oxidative stress and starvation resistance of *dilp* mutants was analysed by monitoring the survival of females on 20 mM paraquat and 1% agar, respectively. None of the *dilp* single mutants or the *dilp2–3* mutants was more resistant to paraquat or starvation treatment (Table 1, Figure S4, Figure S5A). Additionally, *dilp* single mutants did not show increased glycogen or lipid storage, with the exception of *dilp6* mutants, which had slightly increased lipid levels (Figure S5B, S5E). Whole body trehalose levels were increased in *dilp2* mutants, but not changed in *dilp3* or *dilp5* mutants (Figure S5D), consistent with the previous suggestion that stored trehalose levels are specifically regulated by DILP2 [18].

*dilp1–4* mutants were slightly more resistant to paraquat and starvation treatment than controls, and *dilp2–3,5* mutants were highly resistant to oxidative stress, as demonstrated by their increased survival under both paraquat and hydrogen peroxide treatment (Figure S4). However, and in contrast to MNC ablated flies, they were not resistant to starvation (Figure S5), even though they stored more energy in the form of glycogen (Figure S5C) and lipids (Figure S5F). This finding suggests either that lack of DILPs other than DILP2, 3 or 5 is causal for the increased starvation resistance of MNC-ablated flies, or that MNCs mediate starvation resistance independent of DILP function. The latter is consistent with the proposed function of the dARC1 protein in MNCs, which has been suggested to control the behavioural response to starvation, the lack of which might induce starvation resistance [33].

### Adult lifespan

MNC-ablation experiments have suggested a role for DILPs in the determination of lifespan [11],[34]. In particular DILP2 has been proposed by a number of studies to play an important role, because of its transcriptional down-regulation in mutant, long-lived flies [14]–[17]. However, this view has been challenged recently by the finding that RNAi-mediated knock-down of DILP2 is not sufficient to extend lifespan in flies [18].

**Figure 5:**
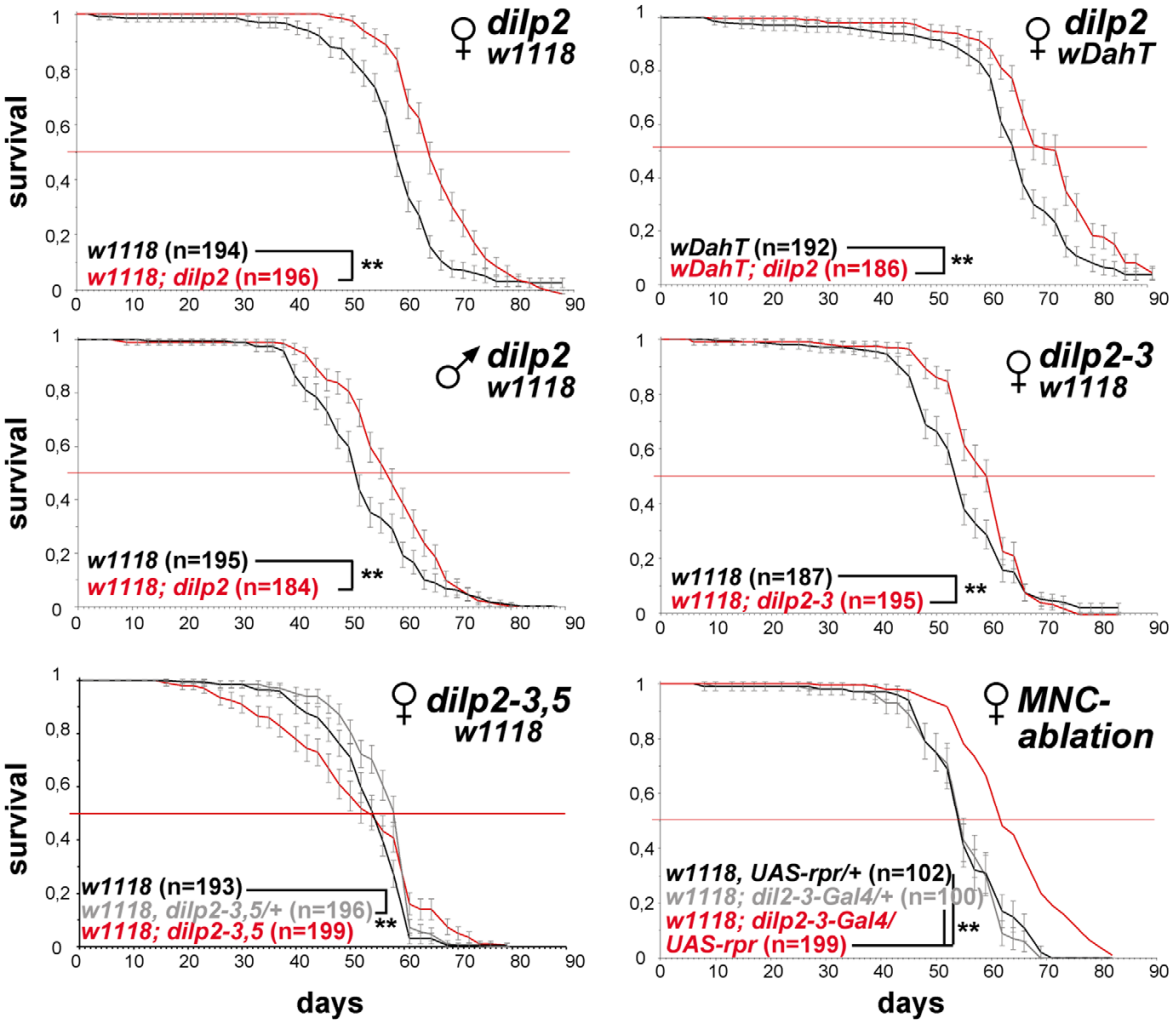
*dilp2* mutant flies are long-lived. Survival curves of *dilp* mutant flies on standard food. Lack-of DILP2 extends median lifespan in two independent genetic backgrounds (*w^1118^* and *w^DahT^)* and in both sexes. Combined knock out of DILP2 and 3 results in increased lifespan. In contrast to MNC-ablated flies, homozygous *w^1118^; dilp2–3,5* mutants do not show increased median lifespan. However, median lifespan is slightly increased in *dilp2–3,5* heterozygous flies. ** p<0.001, log-rank test.

We measured the lifespans of all seven *dilp* null mutants using female flies kept on standard food. We did not observe lifespan-extension in *dilp1, 3, 4, 5, 6* or *7* mutants (Figure S6A). However, in contrast to *dilp2* RNAi hypomorphs, *dilp2* null mutants were significantly longer-lived than controls (Figure 5). An increase between 8% and 13% in median lifespan was observed in four independent trials, two genetic backgrounds and in *dilp2* mutants originating from independent homologous recombination events tested individually or as transheterozygotes (Figure 5 and data not shown). Furthermore, a 9% extension of median lifespan was also observed for *dilp2* mutant males, demonstrating that DILP2 is limiting for lifespan in both sexes. *dilp2–3* mutants were also long-lived, although no more so than *dilp2* mutants (Figure 5). Interestingly, while heterozygous *dilp2–3,5* mutants were slightly long-lived, neither homozygous *dilp2–3,5* mutants nor *dilp1–4* mutants showed an increased median lifespan under standard food conditions (Figure 5, Figure S6A). However, maximum lifespan of homozygous *dilp2–3,5* mutants was increased by 14%, as reported for the demographic aging of flies in which MNC were ablated early during development [35]. These findings might suggest that the strong reduction in insulin signalling in these mutants produced a general decrease in adult viability as well as a slowing down of the increase in death rates with age.

**Figure 6:**
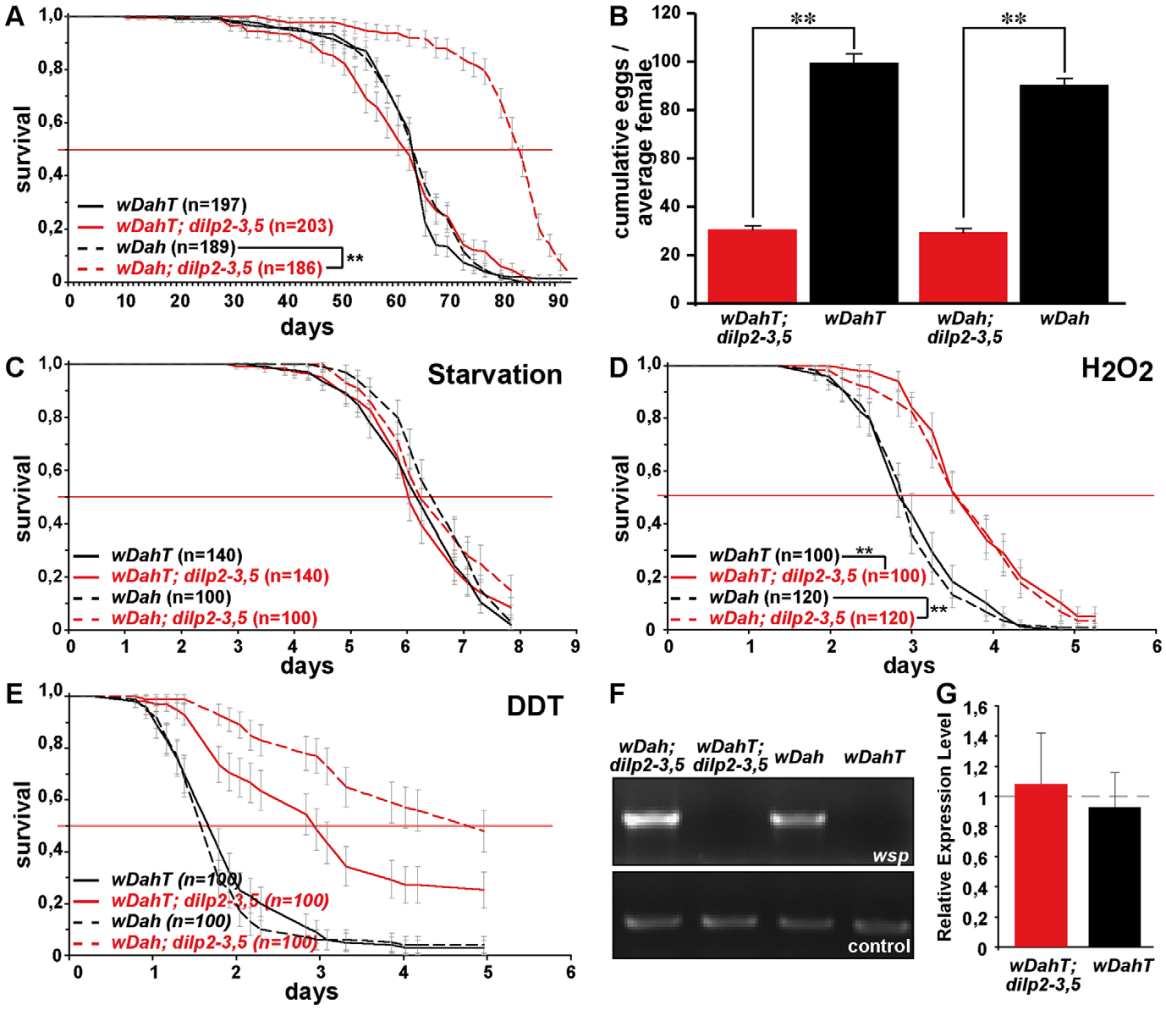
*Wolbachia*-dependent lifespan extension of *dilp2–3,5* mutants is correlated with xenobiotic resistance. *Wolbachia* affects longevity (A) and xenobiotic resistance (E) of *dilp2–3,5* mutants, but not fecundity (B), starvation (C) or oxidative stress resistance (D). (A) Median and maximum lifespan of *w^Dah^;dilp2–3,5* females (83/92,5 days) compared to *w^Dah^* (64,5/76 days) control females is increased by 29% and 22%, respectively. ** p<0.001, log rank test. In contrast, *w^DahT^;dilp2–3,5* (62,5/81 days) only show a small increase in maximum, but not in median lifespan compared to *w^DahT^* (64,5/74 days) females. (B) Index of lifetime fecundity of *dilp2–3,5* females is reduced to 30% but not affected by *Wolbachia* infection. Shown is the cumulative number of eggs laid by an average female. ** p<0.01, Wilcoxon rank sum test. (C–E) Survival of *dilp2–3,5* females with and without *Wolbachia* on (C) 1,5% agarose, (D) 5% hydrogen peroxide and (E) DDT (275 mg/l). ** p<0.01, log rank test. (F) PCR analysis confirms the presence of *Wolbachia* in *w^Dah^* flies and the absence in Tetracycline-treated *w^DahT^* flies. (G) Q-RT-PCR analysis shows that *4E-BP* expression is not affected by *Wolbachia*-infection status. *4E-BP* transcript level in *Wolbachia*- flies was normalised to the corresponding *Wolbachia*+ sample, which by default was set to 1.

**Figure 7:**
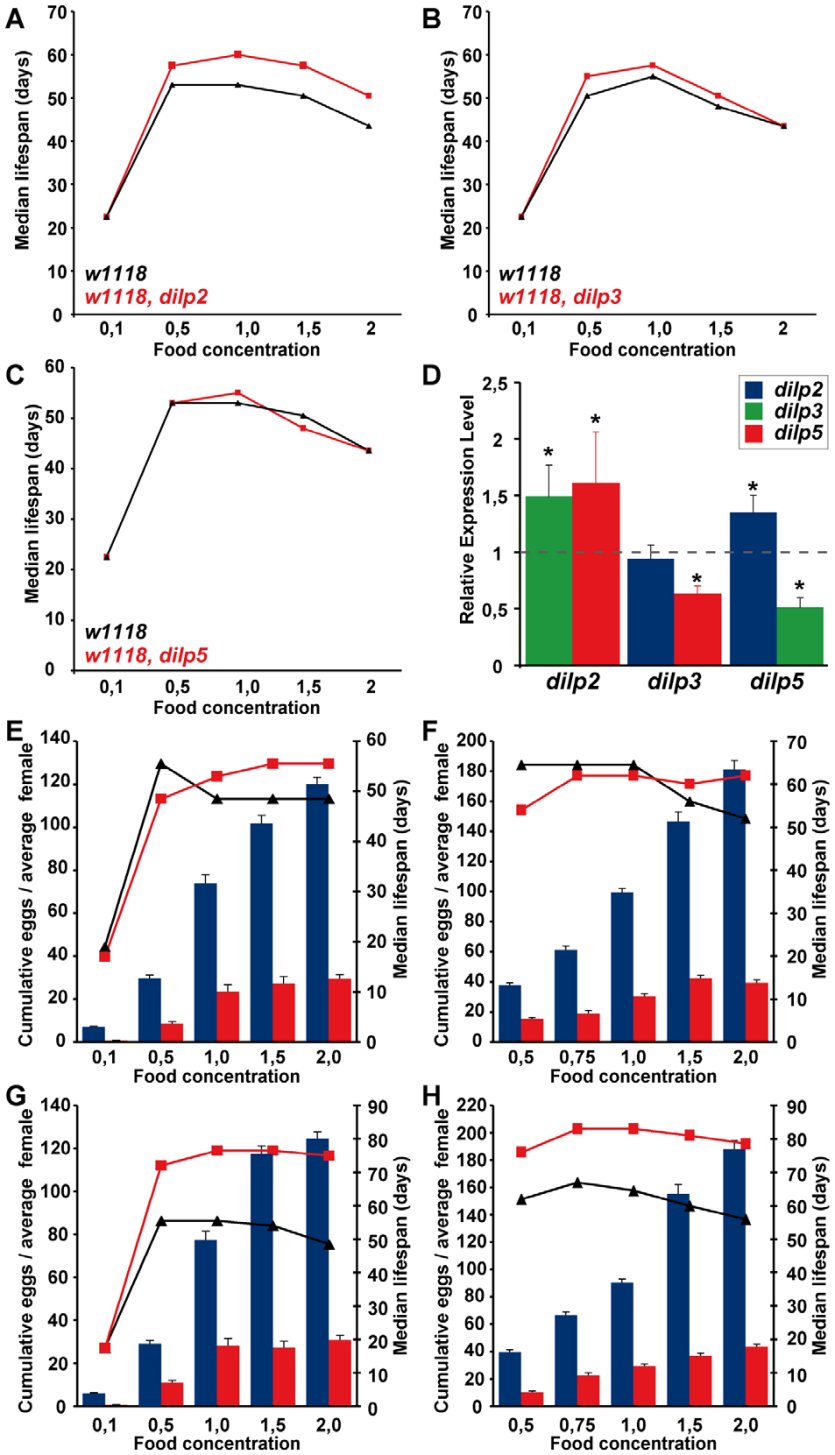
Dietary restriction in *Drosophila* is mediated by DILPs. (A–C) *dilp 2*, *3* or 5 single mutants exhibit a normal response to DR compared to wild type controls. (D) Compensatory regulation among MNC-expressed DILPs on a high yeast diet. Expression levels of DILP2, 3 and 5 were measured by Q-RT-PCR on RNA extracted from heads of 10 day old *dilp* mutant females kept on 2.0x food. * p<0.05. (E–H) In two independent trials *dilp2–3,5* mutants failed to show a normal response to DR. (E,F) *w^DahT^; dilp2–3,5* mutants (*Wolbachia-*). (G,H) *w^Dah^; dilp2–3,5* mutants (*Wolbachia+*) Bars: index of lifetime fecundity±standard error of mean; connected points: median lifespan in days. *dilp2–3,5* mutants in red, *w^DahT^* controls in black/blue.

The intracellular symbiont *Wolbachia pipientis*, a maternally transmitted bacterium, has been shown to modulate longevity in wild type and mutant stocks of *Drosophila* [36],[37] and has recently been suggested to reduce the severity of IIS mutants by increasing IIS downstream of the DInR [38]. Therefore, we decided to test the influence of *Wolbachia* on lifespan and other fitness-related traits of *dilp2–3,5* mutants (Figure 6A*–*6E). Intriguingly, *Wolbachia*-positive *w^Dah^;dilp2–3,5* mutants were extremely long-lived, showing an increase on standard food in both median and maximum lifespan of 29% and 22%, respectively, compared to *w^Dah^* controls (Figure 6A). Lifespan extension was even more pronounced on a high yeast diet with an increase of up to 55% and 27% in medium and maximum lifespan, respectively (Figure 7G). In contrast, *Wolbachia* had no effect on the lifespan of wild type flies (Figure 6A), confirming that lifespan extension of *dilp2–3,5* mutants is the result of a specific interaction between *Wolbachia* and the IIS pathway. However, *Wolbachia* did not affect IIS pathway activity as measured by *4E-BP* expression. Although *4E-BP* was up-regulated in *dilp2–3,5* mutants (Figure 3B), there was no significant difference in *4E-BP* expression between *dilp2–3,5* mutants or wild type flies, respectively, with and without *Wolbachia* (Figure 6G). Some other consequence of insulin signaling must therefore mediate the effects of *Wolbachia*.

Intriguingly, although *Wolbachia* is well known to manipulate the reproductive system of its host, we did not detect significant differences in fecundity between mutants or controls with or without bacterial infection (Figure 6B), and nor did it have an effect on development time (data not shown) or energy storage (Figure S5C, S5F). *Wolbachia* did affect growth, but not by increasing IIS; flies without *Wolbachia* infection had significantly lower body weights than flies carrying the bacterium (Figure 4C), but this effect was seen in wild type and *dilp2–3,5* mutants. *Wolbachia* infection status did not change survival of *dilp2–3,5* mutants under starvation or hydrogen peroxide treatment (Figure 6C and 6D), suggesting that oxidative stress resistance of *w^Dah^;dilp2–3,5* mutants is not causal for their increased lifespan. In contrast, *Wolbachia*-positive *dilp2–3,5* mutants were more resistant to DDT treatment than *Wolbachia*-free *dilp2–3,5* mutants (Figure 6E), which suggests that xenobiotic resistance may contribute to their increased longevity.

### Fecundity

IIS has been implicated in the maintenance of germ-line stem cells (GSC) in *Drosophila* and over-expression of DILP2 suppressed GSC loss in adult females [13]. Consistently, *dilp2* mutant females exhibited a significantly reduced lifetime egg-production (−25%) compared to control flies (Figure S6B). However, fecundity was already reduced in young 3 day old *dilp2* mutant females (data not shown), suggesting that at least part of the phenotype is caused by developmental defects, consistent with results from flies with ablated MNCs [34]. A small decrease in fecundity was also observed in *dilp3* (−22%), *dilp5* (−18%), *dilp2–3* (−27%) and *dilp1–4* (−14%) mutants (Figure S6B). In contrast, egg-production of *dilp2–3,5* mutants was more severely reduced (−69%, Figure 6B), suggesting that DILP2, 3 and 5 can act redundantly in the control of egg-production. Notably, *dilp2–3,5* females are not completely sterile like other strong IIS pathway mutants e.g. homozygous *chico* females [39]. Thus, the finding that *dilp6* mutants exhibited the strongest reduction in fecundity of all *dilp* single mutant females (−46%, Figure S6B) suggests that egg-production is under the combined control of DILPs expressed in MNCs and the fat body. In contrast, fecundity was not significantly reduced in *dilp1, 4* and *7* mutants, the latter contrary to the suggestion that DILP7 may be a *Drosophila* relaxin [7].

### Increased lifespan and reduced fecundity in response to dietary restriction are mediated by DILPs

DR and IIS have been suggested to act via overlapping mechanisms to extend lifespan in flies [19],[22], and the abundance of *dilp5* mRNA is reduced in DR flies [20], suggesting a possible role for DILP5 in the flies' responses to DR. To determine whether DILPs contribute to the DR response, we measured the lifespan of *dilp2, 3* and *5* mutant females (Figure 7A–7C) under DR. The *dilp* single mutants showed a normal DR response, with a peak in lifespan on 1.0x food and the shortest lifespan on 2.0x food observed for both mutants and controls. Except for starvation conditions (0.1x), *dilp2* mutants were long-lived on all food types, suggesting that the lack of DILP2 causes lifespan-extension independent of yeast concentration (Figure 7A). *dilp5* mutants showed a normal DR response, demonstrating that DILP5 is not essential for DR mediated lifespan extension. However, lack of DILP5 may be compensated for by up-regulation of other DILPs on the higher yeast concentrations. We therefore measured DILP transcript levels in the *dilp* mutants on 2.0x food (Figure 7D). Whereas the transcriptional regulation of DILP transcripts in *dilp2* and *dilp3* mutants was similar on low (1.0x) and high (2.0x) yeast concentrations (compare Figure 7D to Figure 3C), in *dilp5* mutants expression of DILP2 was up-regulated on 2.0x food (Figure 7D), suggesting that the lack of diet-dependent DILP5 expression was compensated for by up-regulation of DILP2. Thus, DILPs in the MNC can act redundantly to mediate the organismal response to DR.

To test for redundancy, we measured the DR response of *dilp2–3,5* mutant females in two independent trials using flies with (Figure 7G and 7H) and without *Wolbachia* (Figure 7E and 7F). *Wolbachia*-free *dilp2–3,5* mutants failed to show a normal response to DR. Instead, similar to *chico^1^* mutants, their response was right shifted [22], with the flies shorter-lived compared to controls on low but longer-lived on high yeast concentrations (Figure 7E and 7F). The maximum lifespan of *dilp2–3,5* mutants did not exceed the maximum that was achieved by DR alone, consistent with reduced IIS and DR extending lifespan through the same mechanisms. In addition, whereas wild-type flies showed a strong increase in egg-production between 1x and 2x food (62*–*81%), *Wolbachia*-free *dilp2–3,5* mutants only laid 26% more eggs on the higher yeast concentration (Figure 7E and 7F). Absence of these three DILPs therefore strongly attenuated the response of fecundity to DR. As in wild type flies only DILP5 expression was nutritionally regulated, these results suggest that the normal response to DR is mainly mediated by DILP5.

Interestingly, infection with *Wolbachia* modified the DR response of *dilp2–3,5* mutants. In contrast to *Wolbachia*-free *dilp2–3,5* mutants, these mutants showed extended lifespan at all food concentrations except for starvation, and their maximum lifespan far exceeded that achieved by DR treatment alone (Figure 7G and 7H). In addition, the DR response of the Wolbachia-positive *dilp2–3,5* mutant flies was greatly attenuated but not right shifted. Whereas control flies exhibited a DR-induced lifespan extension of 14*–*20% and an increase in egg production of 108*–*123% between 1x and 2x food, *dilp2–3,5* mutants showed lifespan extension of only 2*–*6% and an increased egg production of 7*–*48%. *Wolbachia* infection status had no effect on the lifespan response to DR of wild type control flies, consistent with previous findings [40]. In conclusion, the DR response of *Wolbachia*-containing *dilp2–3,5* mutants revealed both that these ligands mediate the responses to DR and that reduced IIS extends lifespan through mechanisms that both overlap with those of DR and through additional mechanisms that are independent of those at work in DR.

## Discussion

We conducted a systematic mutational analysis of the 7 *dilp* genes of *Drosophila melanogaster*. Our results show that, while most *dilp* single mutants had only very mild or no obvious phenotypes, combinatorial lack of several DILPs resulted in more severe phenotypes, dependent upon the identity and number of DILPs knocked out, demonstrating that DILPs can act redundantly. Population-genetic theory suggests that newly duplicated gene copies can be evolutionarily unstable due to their functional overlap and that one copy may hence be rapidly lost. However, sufficiently rapid divergence in sequence and function can result in retention of partially redundant genes [23]. We have shown that the 7 *dilp* genes have been evolutionary conserved during the more than 40 million years of evolution of the genus *Drosophila*. Although this time span may seem to be small on a geological timescale, when generation time is taken into account the evolutionary divergence spanned by the genus *Drosophila* exceeds that of the entire mammalian radiation [26].

One hallmark of evolutionarily conserved redundancies is differential expression of redundant genes either spatially or temporally [24],[41]. Consistently, although *dilp* genes have overlapping expression domains, e.g. DILP1, 2, 3 and 5 are co-expressed in MNC during larval development [5],[10], each *dilp* gene also has its own spatio-temporal expression pattern [9] and thereby different DILPs may regulate the same process during different stages of the fly's lifecycle. This is supported by the consecutive activation of DILP2 (first instar), DILP5 (second instar) and DILP3 (mid-late third instar) expression in MNC during development, likely to reflect the requirement for higher DILP levels to support the extensive growth happening especially in later larval stages. Remarkably, only *dilp2* single mutants showed developmental delay and reduced growth, suggesting that DILP3 and DILP5 cannot compensate fully for the lack of DILP2 expression. *dilp2–3,5* triple mutants showed a much more severe delay in larval development and larval growth, suggesting that DILP2, 3, and 5 are the main DILPs controlling growth during larval stages, while the fat body expressed *dilp6* gene has been shown to control growth specifically during pupal development [31]. Thus, individual *dilp* genes use specific enhancer elements resulting in specific spatio-temporal expression, which may explains why the redundant *dilp* genes are stable during the evolution of *Drosophila* flies.

A related explanation for the evolutionary stability of the *dilp* gene family could be that, while DILPs act in part redundantly, they are evolutionarily conserved due to diversification of some of their functions. In support, RNAi-mediated knock-down of DILP2 reduced stored trehalose levels to the same extent as in flies with ablated MNC [18]. We confirmed this finding using the *dilp2* mutants and further showed that *dilp5* and *dilp3* single mutants have normal trehalose levels, suggesting that stored trehalose levels are specifically regulated by DILP2. However, we found no evidence for other specific functions of individual *dilp* genes, which was especially surprising for the evolutionarily more highly conserved DILP4 and DILP7 peptides. DILP7 is the only DILP with *bona fide* orthologues outside the *Drosophila* genus and does not seem to act redundantly with the other DILPs, suggesting that it has a specific and important function. Nevertheless, *dilp7* mutants are viable, have a normal lifespan and are fertile, with no reduction in fecundity. DILP7 has been suggested to be a *Drosophila* relaxin based on the observation that DILP7 neurons project to the female reproductive tract and that silencing of these neurons results in sterile females that exhibit an egg-jamming phenotype [7]. However, our results demonstrate that the sterility of these flies is not due to the lack of DILP7 secretion. Thus, the function of DILP7 and its relatively high evolutionary conservation is currently unclear.

*dilp4* null mutants showed no obvious phenotypes and we found no evidence that DILP4 acts redundantly to the other DILPs. Interestingly, however, by using a DILP4-specific antibody we detected expression of DILP4 in neurons within the brain. The DInR has been shown to be required for axon guidance of photoreceptor-cells during development of the visual system [42], however the ligands mediating this guidance are not known. The neuronal expression may indicate a possible function for DILP4 in axon guidance, which could be experimentally tested in future studies by using the *dilp4* null mutants.

A hallmark of redundant genes is that they are typically cross-regulated by negative feedback [24]. Accordingly, we found up-regulation of DILP3 and DILP5 transcript levels in *dilp2* mutants, consistent with results obtained upon RNAi-mediated knock-down of DILP2 [18]. We also found up-regulation of DILP2 and DILP3 transcript in *dilp5* mutants. While expression of DILP4 and DILP7 was not significantly changed in *dilp2–3,5* mutants (Figure 3B), which suggest that these two DILPs are not part of the MNC negative feedback system, the fat body expressed *dilp6* gene was strongly up-regulated in the triple mutant flies (Figure 3B). Furthermore, the combined lack *of* DILP2, 3, 5 and 6 resulted in lethality, indicating that DILP6 can act redundantly to DILP 2, 3 and 5 and suggesting the existence of a negative feedback system that acts to coordinate DILP expression between MNC in the central nervous system and the peripheral fat body tissue (Figure 3E). The negative feedback system is not restricted to the compensatory regulation of DILP transcript levels, but also involves down-regulation of ImpL2 (Figure 3B), a negative regulator of IIS that directly binds to DILPs and inhibits their function [32].

Interestingly, we found evidence for synergy between the DILPs in the MNCs. DILP2 and DILP5 were both down-regulated in *dilp3* mutants (Figure 3C and 3D), which is consistent with the decreased expression of DILP2 and DILP5 upon RNAi-mediated knock down of DILP3 [34]. In their study Buch et al. interpreted the combined down-regulation as an experimental artefact of the DILP3 RNAi construct due to high sequence homology among the three *dilp* genes [34]. However, the DILP3 RNAi-construct does not contain any off-targets for DILP2 or DILP5 (data not shown) suggesting that the down-regulation of DILP2 and DILP5 is not an experimental artefact but rather that the transcription of these two DILPs is positively regulated by DILP3. Thus, DILP3 might be part of a positive feedback system that acts in an autocrine/paracrine manner to regulate expression of DILPs in MNCs (Figure 3E).

Previous work has shown that the transcript levels of DILP3, but not of DILPs 2 and 5, are reduced in *dFOXO* null mutants [18]. In addition, putative FOXO bindings sites were found to be enriched in the DILP3 promotor region, suggesting that DILP3 levels are regulated by the IIS pathway itself [18]. Interestingly, a similar feed back system has been described for the mammalian analogue of MNC, the pancreatic beta cells, in which insulin regulates its own production [43],[44], suggesting that this feedback system has been evolutionary conserved from insects to mammals.

These findings suggest that autoregulation of DILP3 maintains a necessary minimal level of IIS pathway activity in MNC. In case IIS activity drops below a certain threshold, dFOXO is activated, which in turn upregulates DILP3 expression. The increase in DILP3 levels then causes an additional increase in transcript levels of DILPs 2 and/or 5 (Figure 3E).

Head fat body-specific over-expression of dFOXO has been shown to cause increased lifespan, systemic effects on lipid storage and reduced expression of DILP2 in MNCs [14]. However, molecules mediating the systemic effects of the tissue-specific dFOXO expression have not yet been identified. The findings that DILP6 is both part of a negative feedback system between the fat body and MNCs, which are involved in lifespan and lipid storage regulation, and a direct dFOXO target gene [31] makes DILP6 a good candidate to mediate systemic effects of dFOXO expression. *dilp6* mutant flies were not long-lived, but they will allow testing of whether lifespan extension upon dFOXO over-expression is dependent on DILP6.

In conclusion, DILPs expressed in the MNC and the fat body act redundantly to regulate development, metabolism, reproduction and lifespan and their expression is tightly controlled by both negative and positive feedback mechanisms (Figure 3E). The fly may utilize the presence of redundant DILP copies to downplay stochastic variations in DILP expression or secretion in response to varying external conditions [24]. Synergy of expression, based upon autoregulation through IIS pathway activity, may enable rapid detection of and a systemic response to conditions that lower pathway activity. Because DILPs respond to nutritional changes this may also have helped *Drosophila* flies to adapt to new environments and food sources and thereby facilitated their evolution, generating flies with different feeding habits ranging from generalists like *D. ananassae* to specialists like *D. sechellia*. Although, except for DILP7, *bona fide* orthologues for the other DILPs could not be identified outside the *Drosophilidae*, most animals contain several ILPs, suggesting that, as in *Drosophila*, redundant ILPs may be of evolutionary advantage.

### Specific interaction of *Wolbachia* and IIS in lifespan regulation

*Wolbachia pipientis* are maternally-inherited, obligate intracellular bacteria that are extremely widespread among wild and laboratory *Drosophila* populations [45] and their presence has been associated with parasitic and/or endosymbiontic modification of host fitness-related traits including lifespan [36],[37]. Interestingly, specific interaction between *Wolbachia* strain and host genotype have been demonstrated, e.g. *Wolbachia* can suppress the sterility phenotype of sex-lethal mutants or modify the longevity of a long-lived *Drosophila* strain [37],[46]. Here we show that longevity of *dilp2–3,5* mutant flies is dependent on the presence of a maternally derived factor that can be removed by treatment with Tetracycline, most likely *Wolbachia*. The Tetracycline treatment itself is unlikely to have caused any negative effects because wild type flies with and without *Wolbachia* were phenotypically indistinguishable, except for their different body weight. Furthermore, *dilp2–3,5* mutants had the same median lifespan as control flies in two *Wolbachia*-free genetic backgrounds, which demonstrates that the lack of lifespan extension is not specific to the outbred *w^DahT^* strain. *Wolbachia*-positive *dilp2–3,5* mutants were extremely long-lived and had a prolonged survival under DDT treatment. Increased resistance to xenobiotic compounds has previously been associated with increased longevity [47]. For example, long-lived Little mice, mutant for the GH-releasing hormone receptor gene have been shown to be resistant to xenobiotic toxicity and show concerted up-regulation of xenobiotic detoxification genes [48],[49]. Furthermore, long-lived IIS mutant *C. elegans* and *Drosophila* also show increased expression of genes involved in xenobiotic metabolism [50], suggesting that xenobiotic resistance may contributes to the lifespan extension of *w^Dah^,dilp2–3,5* mutant flies. However, compared to control flies *Wolbachia*-free *dilp2–3,5* mutants also showed increased survival under DDT treatment (Figure 6E), demonstrating that increased xenobiotic resistance alone was not sufficient to increase lifespan, suggesting that in addition other mechanisms contribute to the *Wolbachia*-dependent lifespan extension of *dilp2–3,5* mutants. However, the molecular mechanisms by which *Wolbachia* influences its host are currently unknown.

Notably, lifespan extension of *dilp2* and *dilp2–3* double mutants was not dependent on the presence of *Wolbachia* (Figure 5), demonstrating that *Wolbachia* is not in general essential for lifespan extension due to reduced IIS in *Drosophila*. However, this finding raises the question why *Wolbachia* is essential for lifespan extension in one IIS mutant but not in another. There may exist an optimal range of down-regulation of IIS pathway activity in order to extend lifespan. In contrast to the relative mild phenotypes of *dilp2* or *dilp2–3* mutants, the combined loss of DILP2, 3 and 5 causes severe developmental and metabolic phenotypes, which may be detrimental to the flies, and *Wolbachia* may attenuate the expressivity of the *dilp2–3,5* mutant phenotype. Recently, it has been suggested that *Wolbachia* acts to increase insulin signaling downstream of the DInR and thereby attenuates the phenotype of flies overexpressing a dominant negative DInR [38]. In contrast to these results we did not observe changes in IIS pathway activity when comparing expression of the IIS target 4E-BP in flies with or without *Wolbachia*. In addition, we found no difference in egg-to-adult survival, development time, energy storage, stress resistance or fecundity between *dilp2–3,5* mutants with or without *Wolbachia.* This observation suggests either that lifespan, in contrast to the other traits, is very sensitive to even small changes in IIS activity or that *Wolbachia* mediates lifespan extension of *dilp2–3,5* mutants by another mechanism.

The interaction between *Wolbachia* and its *Drosophila* host are complex and dependent both on the *Wolbachia* strain as well as the genetic background of the fly line. In addition, rapid co-evolution between *Wolbachia* and its host has been demonstrated [51]. In our study we analysed the interaction between IIS and one *Wolbachia* strain in the context of its natural host, the outbred *wDahomey* line. For future studies it will be interesting to determine whether this interaction is specific for *Dahomey* flies and its co-evolved *Wolbachia* strain, or whether other *Wolbachia* strains and/or other *Drosophila* wild type lines have the same effect on IIS.

### DILPs mediate the response to dietary restriction in *Drosophila*

Dietary restriction, the reduced availability of nutrients without malnutrition, extends lifespan in a wide variety of organisms including worms, flies and mammals. However, the underlying molecular pathways mediating the effect of DR on lifespan are still elusive. In *Drosophila*, IIS and DR have been suggested to act through overlapping mechanisms, based on the DR response of *chico* mutants, which are short-lived on low food concentrations but long-lived on high food concentrations [22]. However recently the relevance of IIS for the response to DR in *Drosophila* has been challenged by the finding that flies mutant for the downstream target of IIS the transcription factor dFOXO are short-lived, but respond equally well to DR as control flies [19],[20]. Here we present evidence that DR in *Drosophila* is mediated by the up-stream ligands of IIS, DILPs, expressed in the MNCs. Although *dilp* single mutants responded as strongly to DR as did control flies, the DR response of *dilp2–3,5* mutants was either severely attenuated or completely blocked, depending on the presence of *Wolbachia*, suggesting that DILPs can act redundantly in mediating the response to DR. Additionally, transcript levels of DILP5 were found to be regulated in a diet-dependent manner. Whereas DILP2 and DILP3 transcript levels remained constant across diets, the abundance of DILP5 mRNA was reduced in dietary restricted flies [20], suggesting that, under normal conditions, the response to DR is mediated by DILP5. However, when DILP5 is missing other DILPs may compensate the lack of diet-induced DILP5 expression, consistent with the up-regulation of DILP2 transcript in *dilp5* mutants on high yeast food only. RNAi-mediated knock-down of DILP3 has been shown to reduce DILP5 transcript levels and to block diet-dependent changes in DILP5 transcription and these flies respond normally to DR treatment [20], which we could confirm using the *dilp3* null mutant flies. However, this finding does not exclude a function for DILP5 in the response to DR, as DILP5 peptide is still present and could be regulated on the level of protein stability or secretion.

Thus, ligands of IIS mediate the changes in longevity seen under DR conditions in *Drosophila*, which raises the question why do mutants of the IIS downstream effector dFOXO show a normal response to DR. One explanation could be that dFOXO is involved in the response to DR under normal conditions but in its absence another pathway mediates the life span extension seen upon DR treatment. Fat body specific over-expression of dFOXO extends lifespan in a nutrient dependent manner, which would be consistent with a role of dFOXO in DR [19]. In *C. elegans*, the forkhead transcription factor pha-4, a Foxa orthologue, was shown to be required for lifespan extension under DR [52] and its fly orthologue would therefore be a candidate to mediate DR in the fly, redundant to IIS. The Target of Rapamycin (TOR) pathway has been linked to the determination of lifespan in flies and worms and lifespan-extension by decreased TOR signalling is dependent on nutritional conditions, suggesting a possible link between the TOR pathway and DR [53],[54]. The IIS pathway regulates TOR activity through the protein kinase AKT(PKB), an IIS component downstream of DILPs and Chico but upstream of dFOXO [1]. Thus, in *Drosophila* upstream IIS components such as DILPs and Chico may mediate the response to DR via the TOR pathway but not through the IIS downstream effector dFOXO.

Strong evolutionary conservation of *dilp* gene family membership and sequence differentiation has thus been accompanied by functional differentiation, redundancy and synergy between DILPs, and these features may themselves be of evolutionary advantage.

## Materials and Methods

### Ends-out homologous recombination

*dilp* mutants were generated by ends-out homologous recombination according to the methods described in [30],[55]. All fly stocks are summarized in Table S1. Donor constructs used for targeting *dilp* genes are summarized in Table S2. DNA fragments homologous to approximately 4 kb of *dilp* gene flanking sequences were amplified by PCR and subsequently cloned into the pW25 vector [55]. pw25 was obtained from the *Drosophila* Genomics Resource Center, (Bloomington, Indiana, USA). Long-range PCR was done using Takara LA Taq polymerase (Lonza, UK) on BAC clone DNA as template. BAC clones RP98-7A5 for *dilp1–5* and RP98-32E2 for *dilp7* were obtained from the BACPAC Resource Center (Oakland, California, USA). Primer sequences and restriction sites used for subcloning into pw25 are summarized in Table S3. DILP donor constructs were full-length sequenced and checked for base pair substitutions in gene coding sequences before used to generate transgenic flies. DNA constructs were transformed into the germline of *Drosophila melanogaster* by P-element-mediated germ line transformation using the Best Gene *Drosophila* Embryo Injection Services, (Chino Hills, California, USA). Crosses for ends-out homologous recombination were carried out according to the rapid targeting scheme [56]. Subsequently, the *white^hs^* marker gene was genetically mapped and homologous recombination events were identified by PCR.

In order to generate *dilp2–3,5* and *dilp1–4,5* mutants, homologous recombination was done using flies carrying the *dilp5* donor construct and *hs-FLP, hs-SceI* in the *dilp2–3^1^* and *dilp1–4^1^* mutant background, respectively.

### Generation of *dilp6* mutants

y^1^ P{y[+mDint2] w[BR.E.BR] = SUPor-P}KG04972 [57] flies that carry an KG-element transposon-construct integration in the 5′upstream region of the *dilp6* gene at chromosome X position 2,229,002, corresponding to position -2831 relative to the putative start ATG in *dilp6* exon 2, were obtained from the Bloomington *Drosophila* stock center, Indiana, USA. *dilp6* deletion mutants were generated by conventional P-element imprecise excision resulting in the small *dilp6^41^* deletion and the large *dilp6^68^* deletion. Both *dilp6* alleles contain residual P-element sequence, which impeded the exact mapping of the 3′ breakpoints at the sequence level. By PCR analysis the 3′ breakpoint in *dilp6^41^* could be mapped to the first *dilp6* intron, i.e. *dilp6^41^* mutant flies lack the first *dilp6* exon but contain the full *dilp6* ORF. *dilp6^68^* mutants carry a deletion that covers at least 14 kb of genomic sequence including 5 annotated genes: *dilp6*, *CG33218*, *CG2854*, *CG14050*, *CG34052*.

### Backcrossing and *Wolbachia*

*dilp* mutants were backcrossed for at least ten generations into two different wild-type stocks, the inbred lab strain *w^1118^* and the outbred strain *white DahomeyT* (*w^DahT^*). *dilp6* mutants were only backcrossed into the w^DahT^ background. Naturally, Dahomey flies carry the intracellular bacterium *Wolbachia. w^DahT^* flies were generated by treating *white* Dahomey (w^Dah^) flies with Tetracycline to remove *Wolbachia*. The presence of *Wolbachia* was checked by PCR using a *Wolbachia* specific primer combination (wsp-81F/wsp-691R, Table S3 and Figure 6F). *w^1118^* flies do not contain *Wolbachia* and were therefore not treated with Tetracycline. Unless stated otherwise all experiments were done using *Wolbachia*-free flies. Flies carrying *Wolbachia* were generated by backcrossing *dilp* mutants into the original *w^Dah^* background. All backcrossed stocks were maintained in large numbers in culture bottles on 1,0 SY-A medium at 25°C on a 12L∶12D cycle.

### Generation of experimental flies

Experimental flies were generated by crossing age-matched heterozygous mutants and wild type, heterozygous and homozygous mutant progeny were collected from the same bottle based on eye colour. (Note: as homozygous *dilp5* mutant males could not unambiguously be identified by their eye colour they were not used for experiments.) Eggs were collected during a six-hour time window and the same volume of embryos was transferred to each rearing bottles (1,0 SY-A) ensuring standard larval density. Flies eclosed during a 12 h time window were transferred to new bottles (1,0 SY-A) and left for 48 h to mate (once mated). Subsequently, flies were sorted under brief CO_2_ anaesthesia and transferred to vials. All experiments were conducted at 25°C on a 12 h∶12 h light:dark cycle at constant humidity (65%).

### Lifespan, dietary restriction, and fecundity

For lifespan experiments, flies were maintained in vials at a density of 10 flies per vial on 1.0x SYA medium. Flies were transferred to new vials every two to three days and the number of dead flies was counted. DR experiments were done according to the optimised DR protocol described in [58]. Fly media used for DR experiments are summarized in Table S4. For fecundity assays mated females were kept at a density of 5 females per vial. Eggs were collected during 16*–*20 hour periods during the first 3*–*4 weeks. The number of eggs laid per vial at each time point was counted. Data are reported as the cumulative number of eggs laid per average female ± SEM over the whole period. For DR experiments egg numbers were collected from the lifespan vials.

### Development time and body weight

Development time was analyzed by crossing heterozygous mutant flies and collecting eggs over a period of 3 h. Embryos were allowed to hatch and first instar larvae were hand picked and transferred 50 per vial on standard food. When adult flies started to hatch the number of eclosed homozygous, heterozygous and *w* control flies was counted in regular intervals.

For body weight determination flies were briefly anaesthetized on ice and weighted individually or in pairs on a ME235S analysis balance (Sartorius Mechantronics).

### Stress test and energy storage

For stress tests 20 once mated females per vial were kept on 1x SY-A food for 8*–*10 days before the assay and transferred every other day. Media used for oxidative stress, starvation and DDT assays are summarized in Table S4. For DDT treatment time to knock-down was measured. Organismal lipid, glycogen and trehalose content of 8*–*10 day old females was quantified as described [18],[59].

### Western blot and immunohistochemistry

DILP2 Western blots were done as described [18]. Immunohistochemistry on fly brains of 8*–*10 day old females was done according to the procedures described in [11]. The following primary antibodies were used: anti-DILP2 (rabbit), anti-DILP3 (rabbit), anti-DILP4 (rabbit) and anti-DILP5 (rat). Pictures were taken using an LSM 510 confocal microscope (Zeiss).

### Quantitative RT–PCR

Total RNA was extracted from 25 fly heads or 25 bodies using Trizol (Gibco) according to the protocol of the manufacturer. cDNA was prepared using the Superscript II RT system (Invitrogen) and oligo(dT) primer. Quantitative RT-PCR was performed on a PRISM 7000 sequence detection system using SYBR green master mix (Applied Biosystems) and four independent RNA extractions per genotype. Results were calculated according to the standard curve method and normalized using act primers. Primers used for Q-RT-PCR are summarized in Table S3.

### Data analyses

Lifespan and stress assays were recorded using Excel and were subjected to survival analysis (log rank test) and presented as survival curves. Fecundity data were analysed using the Wilcoxon rank test. Q-RT-PCR, glycogen, trehalose and lipid data were recorded in Excel and analysed using Student's t-test.

## Supporting Information

Figure S1 Amino acid sequence comparison of *Drosophila* DILPs. (A) Amino acid alignments of the 7 DILP pre-propeptides from the 12 sequenced *Drosophila* species. Amino acids identical to the *Drosophila melanogaster* sequence are shaded in yellow. A red cross indicates putative basic cleavage sites; an asterisk marks conserved cysteine residues putatively involved in disulfide bridge formation. (B) Comparison of amino acid sequence identity between the seven DILPs of *Drosophila melanogaster* and *Drosophila grimshawi*. (SP: signal peptide).
(1.10 MB TIF)

Figure S2 Long-range PCR analysis of DILP homologous recombination events. DILP homologous recombination events were tested by long-range PCR on genomic DNA using one primer specific for the *white^hs^* marker gene in combination with one primer specific for the targeted gene region but located outside the respective genomic sequence used in the knock out donor constructs (for details see Text S1). Most targeting events identified by PCR were precise homologous recombinations (see example for DILP3). Arrow marks shorter PCR product in fly line *dilp3^9^*, indicative for an imprecise homologous recombination event. Only DILP knock-out lines with precise homologous recombination events were used for subsequent experiments. C: control, wild type for tested genomic region. M: DNA Marker (HyperLadder I, Biogene). 5′, 3′: amplification of gene region 5′ or 3′ adjacent to the *white^hs^* marker gene integration site, respectively. (Note: In the absence of a *bona fide* PCR template the long range PCR polymerase often produced unspecific PCR products in the wild type controls indicated by an asterisk.
(1.11 MB TIF)

Figure S3 *dilp* mutants generated by homologous recombination are specific DILP protein null alleles. Specific lack of DILP protein expression in *dilp2* mutants (D-F), *dilp3* mutants (G-I), *dilp5* mutants (J-L), *dilp2–3* mutants (M) and *dilp4* mutants (O) is confirmed by immunohistochemistry on adult fly brains using antibodies directed against DILP2 (A, D, G, J, M), DILP3 (B, E, H, K), DILP5 (C, F, I, L) and DILP4 (N-O). (A-M) Magnification of MNCs, (N-O) whole fly brain. Antibody staining on wild type (wt) brains confirms expression of DILP2 (A), 3 (B) and 5 (C) in MNCs. *dilp2* mutants specifically lack expression of DILP2 (D) but not of DILP3 (E) or DILP5 (F). Accordingly, *dilp3* mutants specifically lack expression of DILP3 (H) but not of DILP2 (G) and DILP5 (I) and *dilp5* mutants lack expression of DILP5 (L) but not of DILP2 (J) or DILP3 (K). Note: D/F, G/I and J/L show the same fly brain stained with aDILP2 and aDILP5, respectively. (M) Lack of DILP2 expression in *dilp2-3* double mutants. (N) Using an antibody targeted against DILP4 we found DILP4 to be expressed in neurons throughout the brain. The neuronal antibody staining is specific, as it was absent from *dilp4* mutant brains (O) (Note: a few cells are stained in the *dilp4* null mutant brains, probably a result of unspecific activity of the DILP4 antibody). Scale bar: 20 µm.
(9.79 MB EPS)

Figure S4 Redundant function for DILPs in oxidative stress resistance. Survival of 10-day-old *dilp* mutant female flies on standard food containing 20mM paraquat. Significant paraquat resistance was only observed for *dilp1-4* and *dilp2-3,5* mutants. *dilp2-3,5* mutants also showed increased tolerance against hydrogen peroxide (5% H_2_O_2_). (Note: the same *w^1118^* control was used for *dilp1* and *dilp7*). * p<0.05, ** p<0.01, log rank test.
(0.27 MB TIF)

Figure S5 Starvation survival and energy storage of *dilp* mutants. (A) Survival of *dilp* mutant flies on 1% agarose (starvation). (B) Glycogen content is not changed in *dilp* single mutants. (C) Increased glycogen content of *dilp2-3,5* mutants is independent of their *Wolbachia* infection status. (D) Specific increase of whole fly trehalose content in *dilp2* mutants. (E) Triacylglyceride (TAG) content of *dilp* single mutant flies (*dilp1, 2, 3, 4, 5*: *w^1118^*, *dilp6, 7*: *w^DahT^* background). (F) Increased TAG storage of *dilp2-3,5* mutants is not affected by *Wolbachia*. All experiments in (A-F) were done using once mated 8-10 day old female flies. * p<0.05, ** p<0.01, t-test.
(0.45 MB TIF)

Figure S6 Lifespan and fecundity of *dilp* mutant flies. (A) Survival curves and (B) index of lifetime fecundity of *dilp* mutant female flies (grey) on standard food compared to controls (black) (*dilp1, 2, 3, 4, 5* in *w^1118^ background*; *dilp6, 7, 2-3, 1-4* in *w^DahT^* background). (Note: p<0.07 for *dilp5* mutants) Shown are cumulative eggs laid by an average female. * p<0.05, ** p<0.01, Wilcoxon rank sum test.
(0.31 MB TIF)

Table S1 The fly strains used in this study.
(0.11 MB DOC)

Table S2 Plasmids.
(0.05 MB DOC)

Table S3 PCR oligonucleotide primer.
(0.14 MB DOC)

Table S4 Fly media.
(0.05 MB DOC)

Text S1 Phylogenetic analysis; long-range PCR analysis of *dilp* homologous recombination events; *dilp6* 5′ RACE.
(0.06 MB DOC)
