# Supplementary figures and images for: Molecular Evolution and Functional Characterization of Drosophila Insulin-Like Peptides
Source: PLoS Genet. 2010 Feb 26;6(2):e1000857. doi: 10.1371/journal.pgen.1000857 (PMC2829060; doi:10.1371/journal.pgen.1000857)

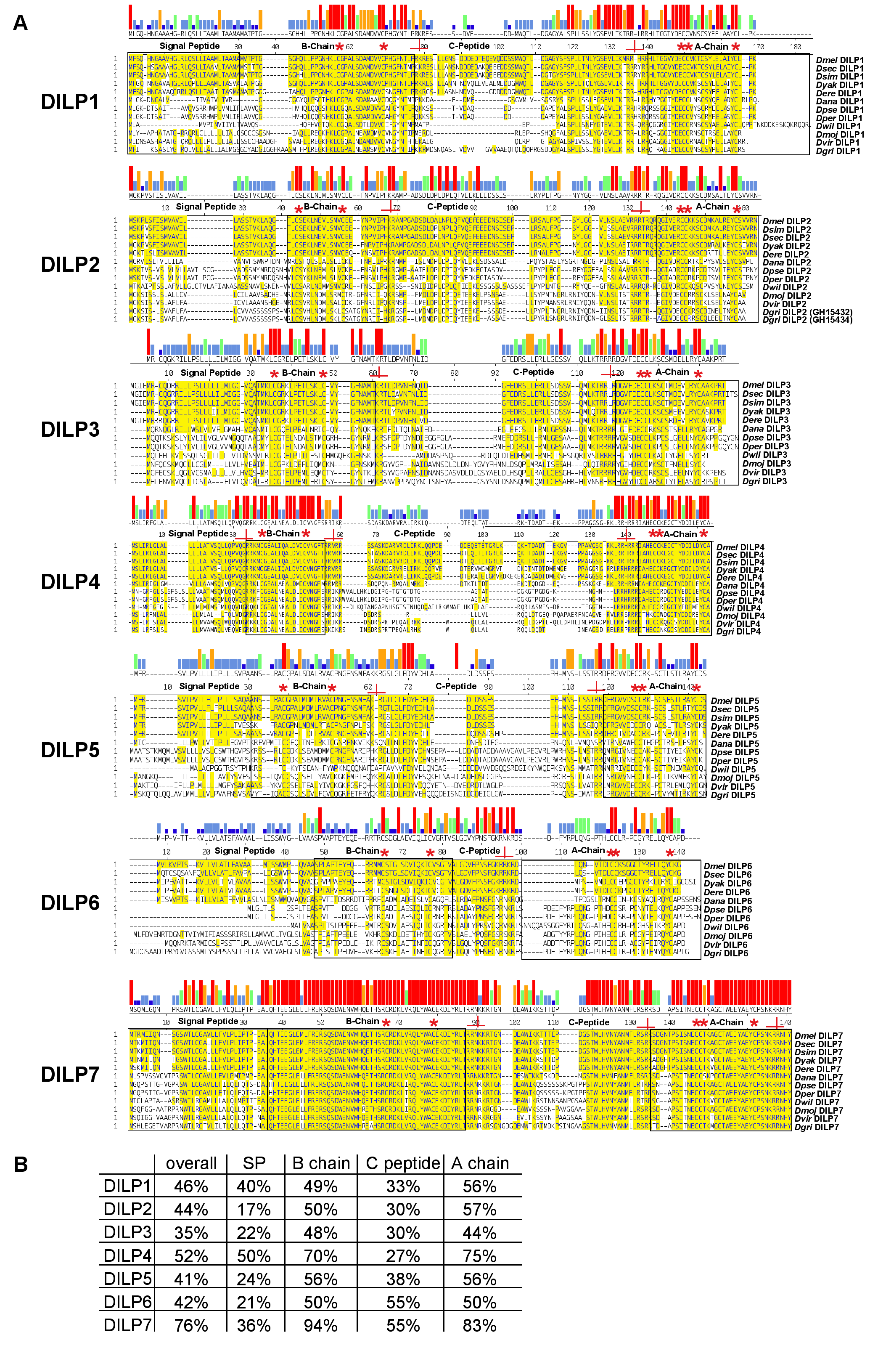

Supplement: Figure S1 — Amino acid sequence comparison of Drosophila DILPs. (A) Amino acid alignments of the 7 DILP pre-propeptides from the 12 sequenced Drosophila species. Amino acids identical to the Drosophila melanogaster sequence are shaded in yellow. A red cross indicates putative basic cleavage sites; an asterisk marks conserved cysteine residues putatively involved in disulfide bridge formation. (B) Comparison of amino acid sequence identity between the seven DILPs of Drosophila melanogaster and Drosophila grimshawi. (SP: signal peptide). (1.10 MB TIF) [file pgen.1000857.s001.tif]

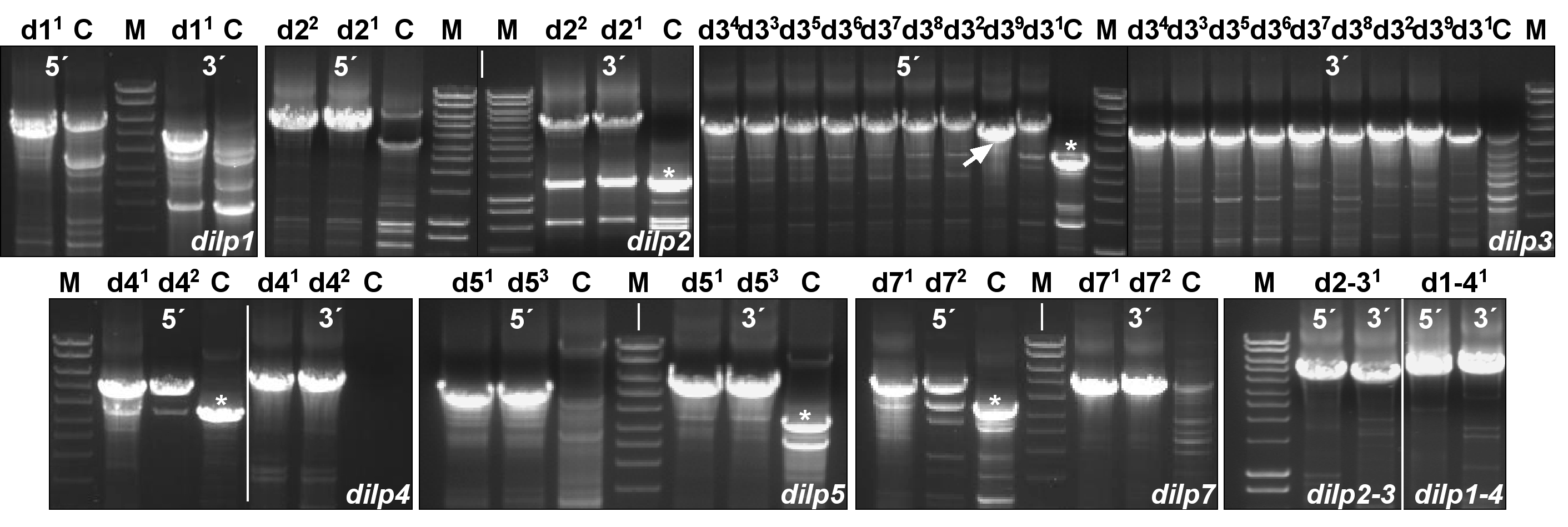

Supplement: Figure S2 — Long-range PCR analysis of DILP homologous recombination events. DILP homologous recombination events were tested by long-range PCR on genomic DNA using one primer specific for the whitehs marker gene in combination with one primer specific for the targeted gene region but located outside the respective genomic sequence used in the knock out donor constructs (for details see Text S1). Most targeting events identified by PCR were precise homologous recombinations (see example for DILP3). Arrow marks shorter PCR product in fly line dilp39, indicative for an imprecise homologous recombination event. Only DILP knock-out lines with precise homologous recombination events were used for subsequent experiments. C: control, wild type for tested genomic region. M: DNA Marker (HyperLadder I, Biogene). 5′, 3′: amplification of gene region 5′ or 3′ adjacent to the whitehs marker gene integration site, respectively. (Note: In the absence of a bona fide PCR template the long range PCR polymerase often produced unspecific PCR products in the wild type controls indicated by an asterisk. (1.11 MB TIF) [file pgen.1000857.s002.tif]

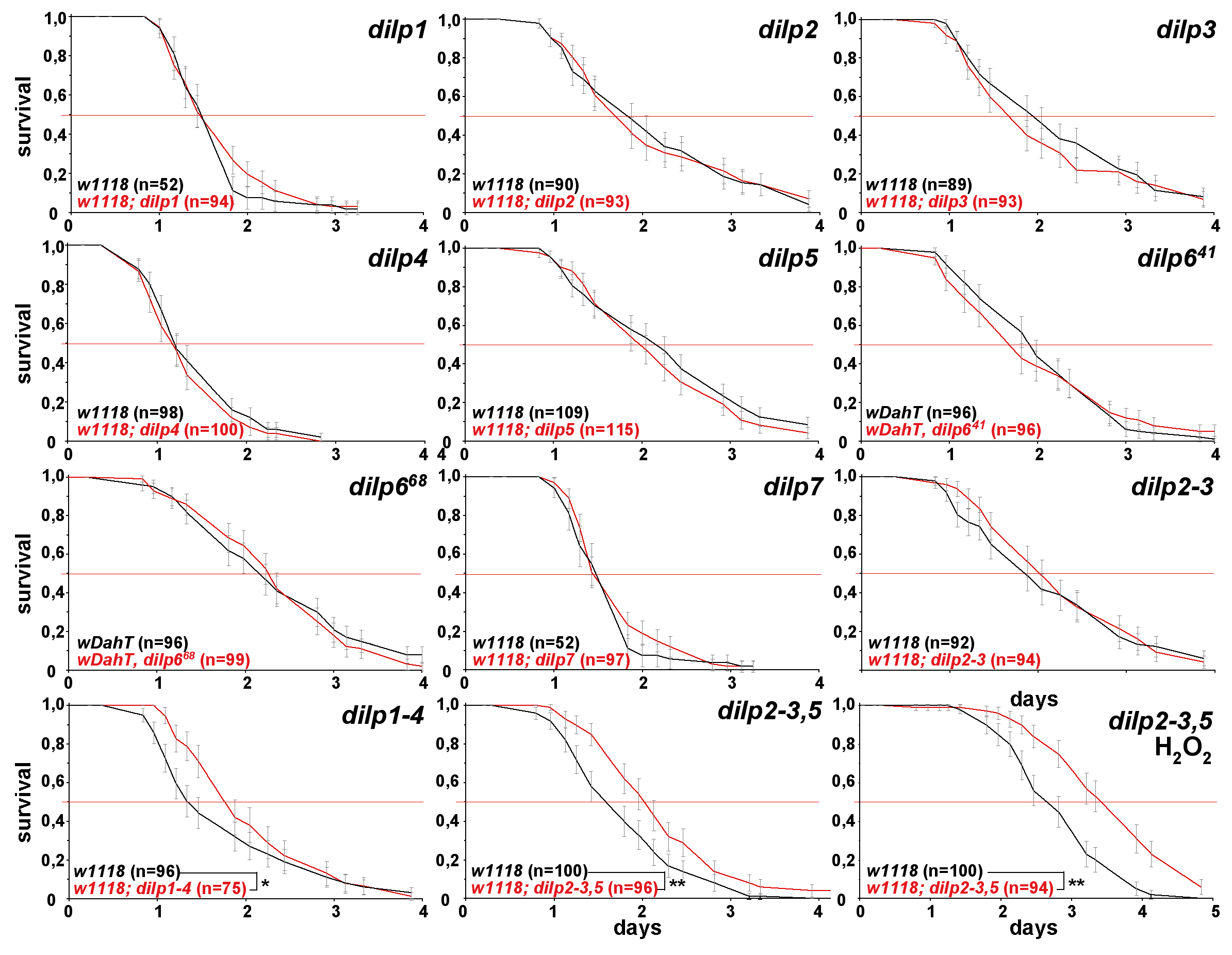

Supplement: Figure S4 — Redundant function for DILPs in oxidative stress resistance. Survival of 10-day-old dilp mutant female flies on standard food containing 20mM paraquat. Significant paraquat resistance was only observed for dilp1-4 and dilp2-3,5 mutants. dilp2-3,5 mutants also showed increased tolerance against hydrogen peroxide (5% H2O2). (Note: the same w1118 control was used for dilp1 and dilp7). * p<0.05, ** p<0.01, log rank test. (0.27 MB TIF) [file pgen.1000857.s004.tif]

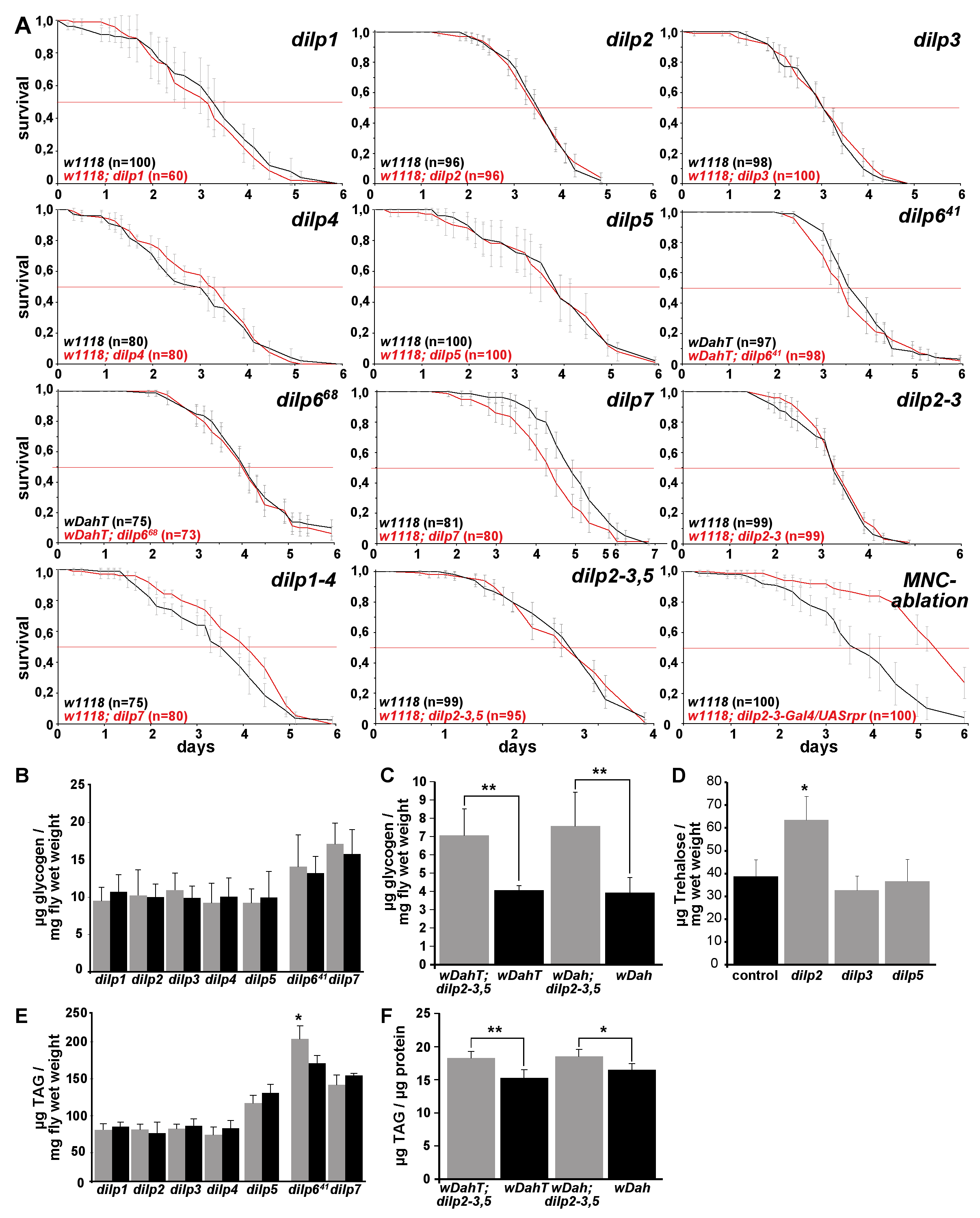

Supplement: Figure S5 — Starvation survival and energy storage of dilp mutants. (A) Survival of dilp mutant flies on 1% agarose (starvation). (B) Glycogen content is not changed in dilp single mutants. (C) Increased glycogen content of dilp2-3,5 mutants is independent of their Wolbachia infection status. (D) Specific increase of whole fly trehalose content in dilp2 mutants. (E) Triacylglyceride (TAG) content of dilp single mutant flies (dilp1, 2, 3, 4, 5: w1118, dilp6, 7: wDahT background). (F) Increased TAG storage of dilp2-3,5 mutants is not affected by Wolbachia. All experiments in (A-F) were done using once mated 8-10 day old female flies. * p<0.05, ** p<0.01, t-test. (0.45 MB TIF) [file pgen.1000857.s005.tif]

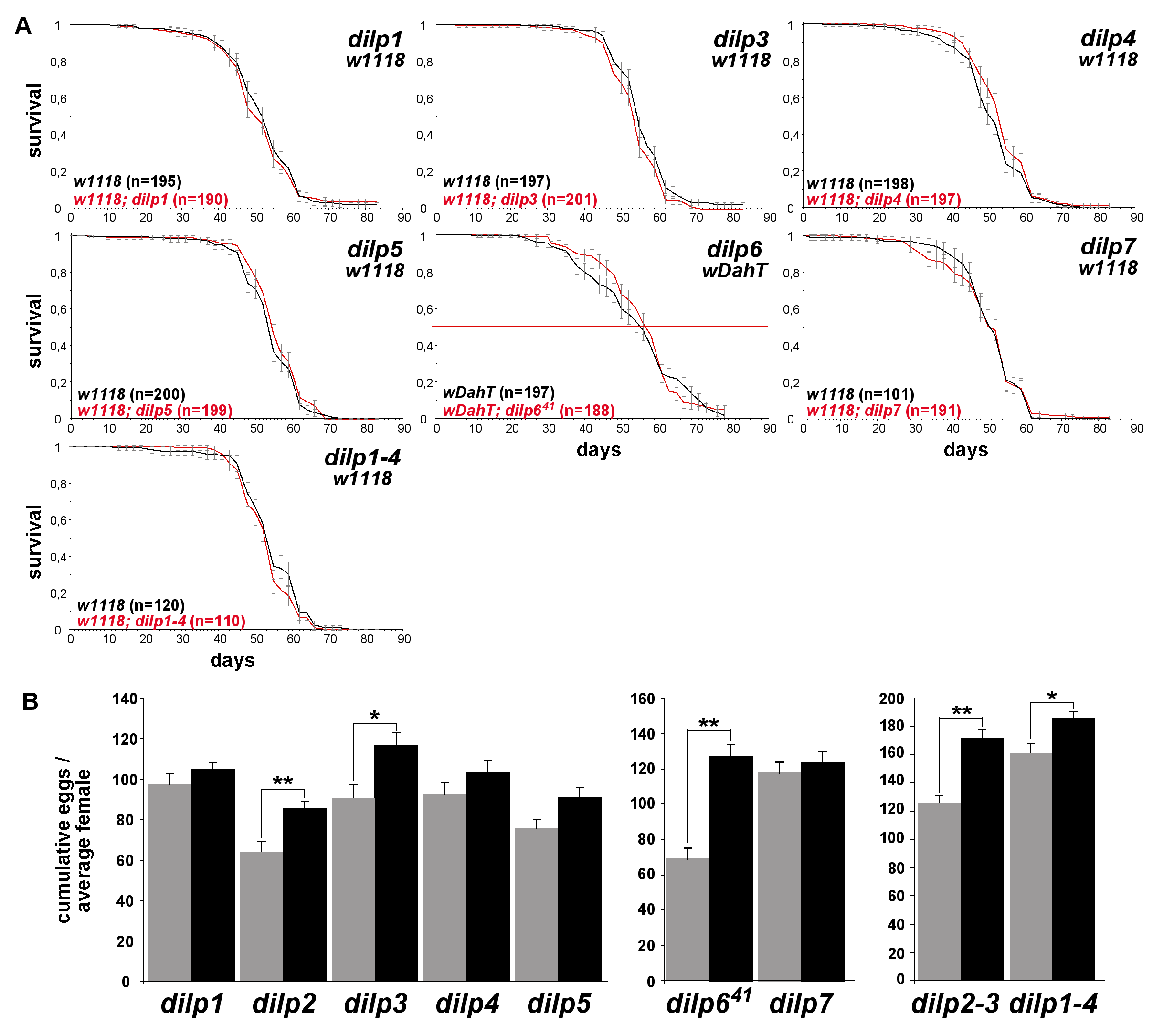

Supplement: Figure S6 — Lifespan and fecundity of dilp mutant flies. (A) Survival curves and (B) index of lifetime fecundity of dilp mutant female flies (grey) on standard food compared to controls (black) (dilp1, 2, 3, 4, 5 in w1118 background; dilp6, 7, 2-3, 1-4 in wDahT background). (Note: p<0.07 for dilp5 mutants) Shown are cumulative eggs laid by an average female. * p<0.05, ** p<0.01, Wilcoxon rank sum test. (0.31 MB TIF) [file pgen.1000857.s006.tif]
