# Supplementary material for: Molecular Evolution and Functional Characterization of Drosophila Insulin-Like Peptides
Source: PLoS Genet. 2010 Feb 26;6(2):e1000857. doi: 10.1371/journal.pgen.1000857 (PMC2829060; doi:10.1371/journal.pgen.1000857)
Supplement: Table S1 — The fly strains used in this study. (0.11 MB DOC) [file pgen.1000857.s007.doc]

**Fly techniques - Table S1:** The following fly strains were used in this study.

| Name (stock number) | Genotype | Genetic background | Reference/Source |
| --- | --- | --- | --- |
| *hs-FLP*  Blo.#6938 | *w^1118^*;P{ry[+t7.2]=70FLP}10 | *-* | [1], Bloomington |
| *hs-FLP,hs-SceI*  Blo.#6934 | *y^1^ w*;*P{ry[+t7.2]=70FLP}11 P{v[+t1.8]=70I-SceI}2B *noc^Sco^*/CyO, *S^2^* | *-* | [1], Bloomington |
| *TM3 Sb* | *w**; *croc^59^ e*/ TM3 *Sb* | *-* |  |
| *dilp*1 ko i/j | *w**; P{w[+mC]=pW25i/j} | *-* | This Study |
| *dilp2* ko g/h | *w**; P{w[+mC]=pW25 g/h#}#/ CyO float | *-* | This Study |
| *dilp3* ko e/f | *w**; P{w[+mC]=pW25 e/f}#5/ CyO float | *-* | This Study |
| *dilp3* ko e/f | *w**; P{w[+mC]=pW25 e/f}#6/ CyO float | *-* | This Study |
| *dilp4* ko k/l | *w**; P{w[+mC]=pW25k/l} | *-* | This Study |
| *dilp5* ko c/d | *w**; P{w[+mC]=pW25c/d}#8/ CyO float | *-* | This Study |
| *dilp7* ko a/b | *w**; P{w[+mC]=pW25a/b} | *-* | This Study |
| *dilp2-3* ko g/f | *w**; P{w[+mC]=pW25g/f} | *-* | This Study |
| *dilp1-4* ko i/l | *w**; P{w[+mC]=pW25i/l} | *-* | This Study |
| KG004972 | *y^1^*, P{y[+mDint2]w[BR.E.BR] =SUPorP}KG004972 | *-* | [2], Bloomington |
| Transposase | FM6, *y**, *w**; MKRS Δ2-3 Sb/TM2 *Ubx* Δ2-3 | *-* |  |
| FM6 | FM6, *y**, *w** | *-* |  |
| *dilp1* mutant | *w**; *dilp1^1^* | *w^1118^, w^DahT^* | This Study |
| *dilp2* mutant | *w**; *dilp2^1^* | *w^1118^, w^DahT^* | This Study |
| *dilp2* mutant | *w**; *dilp2^2^* | *w^1118^, w^DahT^* | This Study |
| *dilp3* mutant | *w**; *dilp3^1^* | *w^1118^, w^DahT^* | This Study |
| *dilp3* mutant | *w**; *dilp3^2^* | *w^1118^, w^DahT^* | This Study |
| *dilp3* mutant | *w**; *dilp3^3^* | *w^1118^, w^DahT^* | This Study |
| *dilp4* mutant | *w**; *dilp4^1^* | *w^1118^, w^DahT^* | This Study |
| *dilp4* mutant | *w**; *dilp4^2^* | *w^1118^, w^DahT^* | This Study |
| *dilp5* mutant | *w**; *dilp5^1^* | *w^1118^, w^DahT^* | This Study |
| *dilp5* mutant | *w**; *dilp5^2^* | *w^1118^, w^DahT^* | This Study |
| *dilp6* mutant | *w**, *y**, *dilp6^41^* | *w^DahT^* | This Study |
| *dilp6* mutant | *w**, *y**, *dilp6^68^* | *w^DahT^* | This Study |
| *dilp7* mutant | *w**, *dilp7^1^* | *w^1118^, w^DahT^* | This Study |
| *dilp2-3* mutant | *w**; *dilp2-3^1^* | *w^1118^, w^DahT^* | This Study |
| *dilp1-4* mutant | *w**; *dilp1-4^1^* | *w^1118^, w^DahT^* | This Study |
| *dilp1-4* mutant | *w**; *dilp1-4^2^* | *w^1118^, w^DahT^* | This Study |
| *dilp2-3, 5* mutant | *w**; *dilp2-3^1^, 5^3^/* TM3 *Sb* | *w^1118^, w^DahT^ w^Dah Wol+^* | This Study |
| *dilp1-4, 5* mutant | *w**; *dilp1-4^1^, 5^4^/* TM3 *Sb* | *w^DahT^* | This Study |
| *dilp2-3, 5,6* mutant | *w**, *y*,6^41^*; *dilp2-3^1^, 5^3^/* TM3 *Sb* | *w^DahT^* | This Study |
| *dilp2-3, 5,6* mutant | *w**, *y*,6^68^*; *dilp2-3^1^, 5^3^/* TM3 *Sb* | *w^DahT^* | This Study |
| *dilp1-4, 5*, 6 mutant | *w**, *y**, *6^41^*; *dilp1-4^1^, 5^4^/* TM3 *Sb* | *w^DahT^* | This Study |
| *dilp1-4, 5*, 6 mutant | *w**, *y**, *6^68^*; *dilp1-4^1^, 5^4^; /* TM3 *Sb* | *w^DahT^* | This Study |
| *dilp2-3, 5, 7* mutant | *w***, 7*^1^; *dilp2-3^1^, 5^3^/* TM3 *Sb* | *w^DahT^* | This Study |
| *dilp1-4, 5, 7* mutant | *w***, 7^1^*; *dilp1-4^1^, 5^4^; /* TM3 *Sb* | *w^DahT^* | This Study |
| *UAS-rpr* | *w^1118^, UAS-rpr* | *w^1118^* | [3] |
| *dilp2-3-Gal4* | *w^1118^; dilp2-3-Gal4* | *w^1118^* | [3] |
| *w^1118^* | Inbred genetic background strain |  | [4] |
| *w^DahomeyT^ (w^DahT^)* | Outbred genetic background strain  Tetracycline treated to remove *Wolbachia* |  | [4] |
| *w^Dahomey Wol+^*  *(w^Dah Wol+^)* | Outbred genetic background strain |  | [4] |

1. Rong YS, Golic KG (2001) A targeted gene knockout in *Drosophila*. Genetics 157: 1307-1312.

2. Bellen HJ, Levis RW, Liao G, He Y, Carlson JW, et al. (2004) The BDGP gene disruption project: single transposon insertions associated with 40% of *Drosophila* genes. Genetics 167: 761-781.

3. Broughton SJ, Piper MD, Ikeya T, Bass TM, Jacobson J, et al. (2005) Longer lifespan, altered metabolism, and stress resistance in *Drosophila* from ablation of cells making insulin-like ligands. Proc Natl Acad Sci U S A 102: 3105-3110.

4. Toivonen JM, Walker GA, Martinez-Diaz P, Bjedov I, Driege Y, et al. (2007) No influence of Indy on lifespan in *Drosophila* after correction for genetic and cytoplasmic background effects. PLoS Genet 3: e95.
