# Supplementary material for: Molecular Evolution and Functional Characterization of Drosophila Insulin-Like Peptides
Source: PLoS Genet. 2010 Feb 26;6(2):e1000857. doi: 10.1371/journal.pgen.1000857 (PMC2829060; doi:10.1371/journal.pgen.1000857)
Supplement: Table S2 — Plasmids. (0.05 MB DOC) [file pgen.1000857.s008.doc]

**Table S2 - Plasmids:**

| Name | Donor construct 5` (primer) | Donor construct 3` (primer) | Reference/Source |
| --- | --- | --- | --- |
| *dilp1* ko (i/j) | i (SOL27/28)  4285 bp | j (SOL29/30)  4312 bp | This Study |
| *dilp2* ko (g/h) | g (SOL23/24)  3991 bp | h (SOL25/26)  3809 bp | This Study |
| *dilp3* ko (e/f) | e (SOL19/20)  4292 bp | f (SOL21/22)  4131 bp | This Study |
| *dilp4* ko (k/l) | k (SOL31/32)  4379 bp | l (SOL33/34)  4507 bp | This Study |
| *dilp5* ko (c/d) | c (SOL15/16)  4242 bp | d (SOL17/18)  4153 bp | This Study |
| *dilp7* ko (a/b) | a (SOL11/12)  4138 bp | b (SOL13/14)  4253 bp | This Study |
| *dilp2-3* ko (g/f) | g (SOL23/24)  3991 bp | f (SOL21/22)  4131 bp | This Study |
| *dilp1-4* ko (i/l) | i (SOL27/28)  4285 bp | l (SOL33/34)  4507 bp | This Study |
| pW25 | - | - | [1], DGRC |

1. Gong WJ, Golic KG (2004) Genomic deletions of th*e Drosophila melanogaste*r Hsp70 genes. Genetics 168: 1467-1476.
