# Supplementary material for: Molecular Evolution and Functional Characterization of Drosophila Insulin-Like Peptides
Source: PLoS Genet. 2010 Feb 26;6(2):e1000857. doi: 10.1371/journal.pgen.1000857 (PMC2829060; doi:10.1371/journal.pgen.1000857)
Supplement: Table S3 — PCR oligonucleotide primer. (0.14 MB DOC) [file pgen.1000857.s009.doc]

**Table S3 - PCR Oligonucleotide Primer:**

| Name | Gene | Sequence | RS | Reference |
| --- | --- | --- | --- | --- |
| SOL8 | *dilp6* | GGGTGTGGCTGAGTGGTGG |  | This Study |
| SOL9 | *dilp6* | ATGGTTCTCAAAGTGCCGACG |  | This Study |
| SOL10 | *dilp6* | GAAAAGGAAAGGAAGCGCCG |  | This Study |
| SOL11 | *dilp7* | TACGTACGTGGCAATGGGCGGCAACAGC | BsiWI | This Study |
| SOL12 | *dilp7* | ATGGCGCGCCATTGGTTAGTTCTTGTGCTCG | AscI | This Study |
| SOL13 | *dilp7* | CTGGTACCATCGACGTTGGATCTGCTTAG | Acc65I | This Study |
| SOL14 | *dilp7* | TAGCGGCCGCCAACCCCCGAAACGCC | NotI | This Study |
| SOL15 | *dilp5* | ATGCGGCCGCAATTGTAATTTGTTAGCAACAG | NotI | This Study |
| SOL16 | *dilp5* | CTGGTACCAATGCTGCGATACCTGGTCC | Acc65I | This Study |
| SOL17 | *dilp5* | ATGGCGCGCCATTGCCTTGCTGGAACTGC | AscI | This Study |
| SOL18 | *dilp5* | AACGTACGCATAATCCCTAAATGCCCAAG | BsiWI | This Study |
| SOL19 | *dilp3* | ATCGTACGGTCTATGAACATCCGCCAGG | BsiWI | This Study |
| SOL20 | *dilp3* | AAGGCGCGCCCAATGACGACAACTGCGATG | AscI | This Study |
| SOL21 | *dilp3* | AAGGTACCCCCGGGGAGTGTGTAGTATG | Acc65I | This Study |
| SOL22 | *dilp3* | TTGCGGCCGCCATGTATTTCCGAGATATTTAG | NotI | This Study |
| SOL23 | *dilp2* | ATCGTACGCATTTGCCAACATTTATTTTCG | BsiWI | This Study |
| SOL24 | *dilp2* | TAGGCGCGCCTCGTAAGAAACTTAAACTGGG | AscI | This Study |
| SOL25 | *dilp2* | GCGGTACCTACTTTATATTATTATTAATTTGGG | Acc65I | This Study |
| SOL26 | *dilp2* | GTATCATTTGGCATGCCCAGCG | SphI | This Study |
| SOL27 | *dilp1* | TTCGTACGTGGAATACCTGAGATGGAG | BsiWI | This Study |
| SOL28 | *dilp1* | TTGGCGCGCCTCTTCTGCAGTCTAGC | AscI | This Study |
| SOL29 | *dilp1* | TAGGTACCGAACACTTGAACATGGCATTC | Acc65I | This Study |
| SOL30 | *dilp1* | TTGCATGCGGTGCCCTTGTTTTC | SphI | This Study |
| SOL31 | *dilp4* | TACGTACGTTCTCCTAACAGCATTCG | BsiWI | This Study |
| SOL32 | *dilp4* | TTGGCGCGCCCTGCAGCATTCCAG | AscI | This Study |
| SOL33 | *dilp4* | ATGGTACCTTTTTGACTTCACGTATTGCG | Acc65I | This Study |
| SOL34 | *dilp4* | AAGCGGCCGCAAAACCCGAACAGC | NotI | This Study |
| SOL39 | *dilp6* | AGACCTCCAGCCCCTTGAACC |  | This Study |
| SOL45 | *dilp3* | CAGAGCCAATATGTGAAGGTGTG |  | This Study |
| SOL54 | pW25 vector | GAGAGGGAGAGTCACAAAACGAA |  | This Study |
| SOL55 | pW25  vector | CGCTGCATGAATTAGCTTGGC |  | This Study |
| SOL68 | *dilp6* | GCGGACTAGCTGCCATCAAATA |  | This Study |
| SOL69 | *dilp6* | GGCGATGGCGATGATGATTATG |  | This Study |
| SOL90 | *dilp3* | GGTGCCCGTGAATCGCTCG |  | This Study |
| SOL92 | *dilp2* | GCCGGATGGAAATGTTTGAGTTG |  | This Study |
| SOL95 | *dilp2* | GCCCTGCGTGGAGTAGAGTGTG |  | This Study |
| SOL96 | *dilp5* | GGCTGTCATTTTGGGCGAAC |  | This Study |
| SOL97 | *dilp5* | CAACAGCAAAGCAGCAACCAGA |  | This Study |
| SOL106 | *dilp4* | GTTTTGCGCTGCGAGTTGTGAA |  | This Study |
| SOL109 | *dilp1* | CTGCAATTTCCTCCTCCACCAGC |  | This Study |
| SOL159 | *dilp6* | GAACAACACATGCCTGGAACACCT |  | This Study |
| SOL160 | *dilp6* | AGGGGGTGGGGTGGTAACTCC |  | This Study |
| SOL138 | *dilp2*  *D. persimilis* | GTCCTGGGGGGTCTTTAGTAATTGG |  | This Study |
| SOL139 | *dilp2*  *D. persimilis* | CGGGTGGCATCTACGACGAGTG |  | This Study |
| SOL167 | *dilp6* | CTTGCAGCACAAATCGGTTACG |  | This Study |
| SOL168 | *dilp7* | GAACGGCGAACAGCAAATGAACTC |  | This Study |
| SOL169 | *dilp7* | CCAAGCGATTATTCGGCTCTATGC |  | This Study |
| *wsp*-691R | *Wolbachia wsp* | AAAAATTAAACGCTACTCCA |  | [1] |
| *wsp*-81F | *Wolbachia wsp* | TGGTCCAATAAGTGATGAAGAAAC |  | [1] |
| dilp2_For  (Q-RT-PCR) | *dilp2* | ATGGTGTGCGAGGAGTATAATCC |  | [2] |
| dilp2_Rev  (Q-RT-PCR) | *dilp2* | TCGGCACCGGGCATG |  | [2] |
| dilp3_For  (Q-RT-PCR) | *dilp3* | AGAGAACTTTGGACCCCGTGA A |  | [2] |
| dilp3_Rev  (Q-RT-PCR) | *dilp3* | TGAACCGAACTATCACTCAACAGT CT |  | [2] |
| dilp4_For  (Q-RT-PCR) | *dilp4* | GCGGAGCAGTCGTCTAAGGA |  | This study |
| dilp4_Rev  (Q-RT-PCR) | *dilp4* | TCATCCGGCTGCTGTAGCTT |  | This study |
| dilp5_For  (Q-RT-PCR) | *dilp5* | GAGGCACCTTGGGCCTATTC |  | [2] |
| dilp5_Rev  (Q-RT-PCR) | *dilp5* | CATGTGGTGAGATTCGGAGCTA |  | [2] |
| dilp6_For  (Q-RT-PCR) | *dilp6* | CGATGTATTTCCCAACAGTTTCG |  | This study |
| dilp6_Rev  (Q-RT-PCR) | *dilp6* | AAATCGGTTACGTTCTGCAAGTC |  | This study |
| dilp7_For  (Q-RT-PCR) | *dilp7* | CAAAAAGAGGACGGGCAATG |  | This study |
| dilp7_Rev  (Q-RT-PCR) | *dilp7* | GCCATCAGGTTCCGTGGTT |  | This study |
| 4E-BP-For  (Q-RT-PCR) | *thor* | CACTCCTGGAGGCACCA |  |  |
| 4E-BP-Rev  (Q-RT-PCR) | *thor* | GAGTTCCCCTCAGCAAGCAA |  |  |
| act-5c-For  (Q-RT-PCR) | *ac-5ct* | CAC ACC AAA TCT TAC AAA ATG TGT |  | [2] |
| act-5c-Rev  (Q-RT-PCR) | *act-5c* | AAT CCG GCC TTG CAC ATG |  | [2] |

1. Toivonen JM, Walker GA, Martinez-Diaz P, Bjedov I, Driege Y, et al. (2007) No influence of Indy on lifespan in Drosophila after correction for genetic and cytoplasmic background effects. PLoS Genet 3: e95.

2. Broughton SJ, Piper MD, Ikeya T, Bass TM, Jacobson J, et al. (2005) Longer lifespan, altered metabolism, and stress resistance in Drosophila from ablation of cells making insulin-like ligands. Proc Natl Acad Sci U S A 102: 3105-3110.
