# Supplementary material for: Molecular Evolution and Functional Characterization of Drosophila Insulin-Like Peptides
Source: PLoS Genet. 2010 Feb 26;6(2):e1000857. doi: 10.1371/journal.pgen.1000857 (PMC2829060; doi:10.1371/journal.pgen.1000857)
Supplement: Table S4 — Fly media. (0.05 MB DOC) [file pgen.1000857.s010.doc]

**Table S4 - Fly media:**

| Food Type | Sugar (g/l) | Yeast (g/l) | Agar (g/l) | Water (ml) | Propionic acid (ml) | Nipagin (ml) |
| --- | --- | --- | --- | --- | --- | --- |
| SY-A 0,10 | 50 | 10 | 15 | 917 | 3 | 30 |
| SY-A 0,50 | 50 | 50 | 15 | 912 | 3 | 30 |
| SY-A 0,75 | 50 | 75 | 15 | 899 | 3 | 30 |
| SY-A 1,00  (Standard) | 50 | 100 | 15 | 886 | 3 | 30 |
| SY-A 1,50 | 50 | 150 | 15 | 860 | 3 | 30 |
| SY-A 2,00 | 50 | 200 | 15 | 834 | 3 | 30 |
| Starvation | - | - | 10 | 1000 | - | - |
| Paraquat  20 mM | 50 | 100 | 15 | 886 | 3 | 30 |
| Hydrogen peroxide 5% (5%) | 50 | - | 15 | 1000 | - | - |
| DDT  275 mg/l | 50 | 100 | 15 | 954 | 3 | 30 |

Yeast (brewers yeast #903312, MP Biomedicals, London, UK); sugar (Tate & Lyle Sugars, London, UK); agar and propionic acid (Sigma, Dorset, UK); Nipagin M (methyl 4-hydroxybenzoate, Clariant UK Ltd, Pontypridd, UK); paraquat (Methyl viologen dichloride hydrate, Sigma-Aldrich, UK); hydrogen peroxide (Sigma-Aldrich, UK); DDT (o,p′ & p,p′, PS74 Supelco, Sigma-Aldrich, UK).
