# Supplementary material for: Molecular Evolution and Functional Characterization of Drosophila Insulin-Like Peptides
Source: PLoS Genet. 2010 Feb 26;6(2):e1000857. doi: 10.1371/journal.pgen.1000857 (PMC2829060; doi:10.1371/journal.pgen.1000857)
Supplement: Text S1 — Phylogenetic analysis; long-range PCR analysis of dilp homologous recombination events; dilp6 5′ RACE. (0.06 MB DOC) [file pgen.1000857.s011.doc]

**Supplemental Material and Methods**

**Phylogenetic analysis.** Homology searches identified translated DILP sequences from Flybase release 1.2 (*D. ananassae, D. erecta, D. grimshawi, D. mojavensis, D. persimilis, D. sechellia, D. simulans, D. virilis, D. wilistoni, D. yakuba*), release 2.2 (*D. pseudoobscura*) and release 5.9 (*D. melanogaster*). Many of the DILP sequences were found to be truncated, but could be extended through alignment of the reverse transcribed amino acid sequence to the genome sequence. *dilp2* from *D. persimilis* was not covered by the genome sequence and was therefore cloned by PCR on genomic DNA prepared from *D. persimilis* flies (UC San Diego *Drosophila* Species Stock Center) using primers SOL138 and SOL139 (Table S3). The corresponding DILP2 peptide sequence was found to be identical to the published *D. pseudoobscura* DILP2 sequence (Flybase GA20861-PA). Alignments using clustal W [1] were constructed for each DILP orthologue groups and for each of the seven paralogous DILPs from *D. melanogaster*. A full alignment of all DILPs from all species was constructed by hand by merging the alignments for each DILP orthologue group, using the alignment generated for the paralogous *D. melanogaster* DILPs as a guide. The evolutionary history of the DILPs within the 12 Drosophila species was inferred using the Neighbour-Joining method [2], as implemented in the MEGA software package [3]. Evolutionary distances between the DILP amino acid sequences were calculated using the JTT method [4], only excluding alignment gaps in a pairwise sequence comparison manner. Branch support values within the inferred tree were assessed via bootstrap replicates [5].

**Long-range PCR analysis of *dilp* homologous recombination events.** Genomic DNA of putative *dilp* homologous recombination events was purified using the DNeasy Blood and Tissue kit (Qiagen). Long-range PCR was done using Takara LA Taq polymerase (Lonza, UK) and the following primer combinations: *dilp1*: 5´ SOL109/55, 3´ SOL54/32; *dilp2*: 5´ SOL92/55, 3´ SOL95/54; *dilp3*: 5´ SOL55/45, 3´ SOL54/90; *dilp4*: 5´ SOL29/55, 3´ SOL106/54; *dilp5*: 5´ SOL97/54, 3´ SOL96/55; *dilp7*: 5´ SOL168/55, 3´ SOL169/54, *dilp2-3*: 5´ SOL92/55, 3´ SOL54/90 and *dilp1-4*: 5´ SOL109/55, 3´ SOL106/54 (Table S3).

***dilp6* 5´RACE**. Total RNA was prepared from adult males of *dilp6^41^* mutants, *dilp6^68^* mutants and *wDahT* wild type controls. The 5´RACE was done using the FirstChoice® RLM-RACE Kit (Ambion) according to the instructions of the manufacturer. SOL8 was used in combination with the outer primer for the first round, SOL167 in combination with the inner primer in the second round of the nested PCR. Specific PCR products were only obtained from *dilp6^41^* mutants and *wDahT* control flies, subcloned in pCRII-Topo vector (Invitrogen) and sequenced.

The 5` RACE on wild type RNA showed that *dilp6* exon 1 starts 407 bp further 5´ than previously annotated (Flybase *D. melanogaster* Genome release 5.17: 2227353-2228126).

*dilp6* exon1 wild type (based on two independent 5´RACE reactions): aaaagcgttttcaattccaagccagtcgcaacgacagcggaataccgctacaattcgcactctcagtcgactgccctctcactcactcacctccttctcctgcggcccgagacgtatacccgcactcttctgttcctgccacaatacttcacgcgatgaaacaacaactggcgggcgcagcagcagcagaaaacgcagcagccgcaacagcagtcgaaaacgcagcagacgcaccagaagttaaccacttgctcgattttccacactccgaacgccaatcgattttaaaaggacattgatcgaacgtcaatcgccaaactcacggatatttatcaattcgcctttttttttgcacgtaaaaaccctaacaaacatttaaaaaaaaaaaaacccaaacgaatcccaccagaaaacgatagttaccccttattattagtcgttttgttttgttttttcgtcaattgattgtgattttgaaactgtgtgatcgcaaaagaaaatctgcaaattagcaaaaaaagaaagttccttaagcaagtgataatccgatagaaactctaaagacagaaaaggaaaggaagcgccggagaaaagagacaaaatatacacgaaactaaaagctcccaatatcagcacctatatatattttatagtccacgaaatcaaacaaaacataaatatcataagtcactcttagtcattcgtcatcgaggactacactactaaggaaactaattaatactacaaaaacaaggattaaacggcatgcaaac

In *dilp6^41^* mutants two different 5`RACE products were identified, both of them fusion transcripts that lacked the first *dilp6* exon but still contained the full *dilp6* ORF (see below). The first transcript, in which a part of the remaining KG element was spliced to *dilp6* exon 2, contained an ORF encoding part of the transposase peptide starting 5´ and overlapping with the *dilp6* ORF.

The second transcript, a fusion between *dilp6* exon 2 and genomic sequence close to the adjacent *phl* gene, also contained a short ORF that terminated immediately upstream of the *dilp6* ORF. Thus, the translation of these ectopic ORFs upstream of the *dilp6* ORF in the fusion transcripts might interfere with the translation of the DILP6 peptide.

*dilp6^41^* transcript 1:

cgcggatccgaacactgcgtttgctggctttgatgaaacagtgcacgtttgcttgttgagaggaaaggttgtgtgcggacgaatttttttttgaaaacattaacccttacgtggaataaaaaaaa*ATGAAATATTGCAAATTTTGCTGCAAAGCTGTGACTGGAGTAAAATTAATTCACGTGCCGAAGTGTGCTATTAAGAGAAAATTGTGGGAGCAGAGCCTTGGGTGCAGCCTTGGTGAAAACTCCCAAATTTGTGATACCCACTTTAATGATTCGCAGTGGAAGGCTGCACCTGCAAAAGGTCAGACATTTAAAAGGAGGCGACTCAACGCAGATGCCGTACCTAGTAAAGTGATAGAGCCTGAACCAGAAAAGATAAAAGAAGGCTATACCAGTGGGAGTACACAAACAGA****ATGGTTCTCAAAGTGCCGACGTCCAAAGTCCTGCTAG*TCCTGGCCACCTTGTTCGCCGTGGCGGCGATGATCAGCAGCTGGATGCCCCAGGTGGCGGCCAGTCCGCTCGCACCCACGGAATACGAACAGAGACGCATGATGTGCTCCACCGGCCTCAGCGATGTGATACAGAAGATATGCGTAAGCGGAACGGTGGCCCTTGGCGATGTATTTCCCAACAGTTTCGGGAAGCGCAGGAAGCGCGACTTGCAGAACGTAACCGATTTGTGCTGCAAG** (transposase ORF in capitalized italic, dilp6 ORF in capitalized bold).

Transposase peptide encoded by the ectopic ORF in *dilp6^41^* transcript 1:

MKYCKFCCKAVTGVKLIHVPKCAIKRKLWEQSLGCSLGENSQICDTHFNDSQWKAAPAKGQTFKRRRLNADAVPSKVIEPEPEKIKEGYTSGSTQTewfskcrrpksc.

*dilp6^41^* transcript 2:

atcaactaaccatatacaagtgcaggtggtagtgatggaagaaacatag*ATGGGTTTGATTCAAGTGGCAGCCCCGACACAAGCGTCGGGTTCCGGCAAATCACCAATAGATGGCGTGCATAGGCGCGAAATTTGTATTAAATGCCACTTTTGGTCGCTTGAAATGATACAAATAAATGAAACCATTATTATTATTTTAAAACGATTTCAACACGATCTTTGA*a**ATGGTTCTCAAAGTGCCGACGTCCAAAGTCCTGCTAGTCCTGGCCACCTTGTTCGCCGTGGCGGCGATGATCAGCAGCTGGATGCCCCAGGTGGCGGCCAGTCCGCTCGCACCCACGGAATACGAACAGAGACGCATGATGTGCTCCACCGGCCTCAGCGATGTGATACAGAAGATATGCGTAAGCGGAACGGTGGCCCTTGGCGATGTATTTC** (ectopic ORF in capitalized italic, dilp6 ORF in capitalized bold).

Supplemental References

1. Thompson JD, Higgins DG, Gibson TJ (1994) CLUSTAL W: improving the sensitivity of progressive multiple sequence alignment through sequence weighting, position-specific gap penalties and weight matrix choice. Nucleic Acids Res 22: 4673-4680.

2. Saitou N, Nei M (1987) The neighbor-joining method: a new method for reconstructing phylogenetic trees. Mol Biol Evol 4: 406-425.

3. Tamura K, Dudley J, Nei M, Kumar S (2007) MEGA4: Molecular Evolutionary Genetics Analysis (MEGA) software version 4.0. Mol Biol Evol 24: 1596-1599.

4. Jones DT, Taylor WR, Thornton JM (1992) The rapid generation of mutation data matrices from protein sequences. Comput Appl Biosci 8: 275-282.

5. Felsenstein J (1985) Confidence limits on phylogenies: An approach using the bootstrap. Evolution 39: 783-791.
